# The Combined Effect of Mindfulness and Physical Activity on Stress: A Systematic Review and Meta-Analysis of Randomized Controlled Trials

**DOI:** 10.3390/bs16091630

**Published:** 2026-09-11

**Authors:** Cong Liu, Wenfeng Tan, Yuyan Liu, Yuyang Nie, Wenxue Ma, Jinqi Liang, Shuqi Zeng, Fan Wu, Kangli Du, Lijia Hou

**Affiliations:** 1 College of Future Education, Beijing Normal University, Zhuhai 519087, China; congliu@bnu.edu.cn (C.L.);; 2 College of Physical Education and Sport, Beijing Normal University, Beijing 100875, China; 202522070119@mail.bnu.edu.cn (W.T.);

**Keywords:** mindfulness, physical activity, stress, meta-analysis

## Abstract

Background: Interventions combining mindfulness and physical activity are increasingly used for stress management, but their average efficacy and incremental benefit over either component alone remain uncertain. Objective: This study aimed to evaluate combined mindfulness–physical activity interventions for stress or stress-related outcomes relative to passive or general control conditions, mindfulness alone, and physical activity alone, and to explore potential sources of heterogeneity. Methods: Following PRISMA 2020, eight electronic databases were searched through November 2025. Two reviewers independently screened records. Data extraction and the initial risk-of-bias assessment were performed by the first author and checked in full by a second reviewer. Risk of bias was assessed using RoB 1.0. Certainty of evidence was assessed using the GRADE approach. Hedges’ *g* values were synthesised in three separate comparator-specific meta-analyses using restricted maximum-likelihood random-effects models with Knapp–Hartung adjustment. Results: Fourteen randomised controlled trials involving 872 randomised participants were included in the systematic review, and 13 contributed to quantitative synthesis. Compared with passive or general control conditions, combined interventions produced a statistically significant average effect favouring the combined intervention (*k* = 12; Hedges’ *g* = −0.74, 95% CI −1.21 to −0.27; *p* = 0.006; *I*^2^ = 83.1%; 95% prediction interval −2.27 to 0.79). The combined intervention was not statistically superior to mindfulness alone (*k* = 2; Hedges’ *g* = 0.49, 95% CI −1.71 to 2.69) or physical activity alone (*k* = 3; Hedges’ *g* = 0.01, 95% CI −0.50 to 0.52), although both comparisons were imprecise. No formal subgroup test identified a statistically significant moderator. Excluding the most extreme estimate reduced the pooled control-comparison effect to −0.55 (95% CI −0.79 to −0.31) and *I*^2^ to 27.9%. Conclusions: Combined mindfulness and physical activity interventions may reduce stress or stress-related psychological outcomes relative to passive or general controls. The current evidence is insufficient to determine whether they provide additional benefit over well-delivered single-component interventions. Substantial heterogeneity and the small number of active-comparator trials warrant cautious interpretation.

## 1. Introduction

Psychological stress is a multidimensional response to demands that are appraised as exceeding an individual’s adaptive resources. When persistent, it can impair well-being and daily functioning and contribute to the development or progression of mental and physical illness (Cohen et al., 2007; Kivimäki & Steptoe, 2018). Because stress-related problems occur across both clinical and nonclinical populations, accessible non-pharmacological strategies that can address psychological symptoms while also supporting physical health are of substantial public-health interest.

Mindfulness-based interventions and physical activity are two widely studied approaches. Meditation programmes can improve several stress-related outcomes, although effects vary by programme, population, comparator, and outcome (Goyal et al., 2014). Physical activity interventions likewise improve depression, anxiety, and psychological distress across diverse adult populations (Singh et al., 2023). The two approaches may act through partly distinct but potentially complementary pathways. Mindfulness training emphasises present-moment attention, acceptance, and reduced reactivity to internal experiences, whereas physical activity may influence affect, sleep, behavioural activation, stress physiology, and broader physical health (Tang et al., 2015; Pedersen & Saltin, 2015). These complementary targets provide a plausible rationale for combining the two components, but a theoretical rationale alone does not establish that a combined intervention is more effective than either component delivered well on its own.

Combined mindfulness–physical activity interventions are not a single standardised treatment. They include programmes in which mindfulness instructions are embedded within movement, as well as programmes in which meditation and physical activity are delivered as distinguishable components. The mindfulness-related element may range from structured mindfulness-based stress reduction to brief meditation, mindful walking, mindful self-hypnosis, or mindfulness cueing during yoga or Tai Chi; the physical activity element may involve walking, stretching, resistance training, yoga, Tai Chi, or mixed exercise. Importantly, movement modalities such as yoga, Tai Chi, and Qigong should not be assumed to contain a mindfulness component unless the original intervention explicitly describes mindfulness- or meditation-related instructions. This conceptual and procedural heterogeneity complicates both intervention classification and interpretation of pooled effects.

Previous reviews have addressed related but not identical questions. Evidence has been synthesised for mind–body exercise and perceived stress (Zou et al., 2018); for complex interventions combining physical activity and mindfulness across broad mental-health and well-being outcomes (Remskar et al., 2024); for combined interventions targeting depressive symptoms (Huang et al., 2024); and for mindful movement interventions addressing anxiety and depression in university students (Xu et al., 2025). Taken together, these reviews suggest potential benefits, but do not resolve three issues central to stress management: whether combined interventions improve stress or stress-related psychological outcomes relative to passive or general control conditions; whether they provide incremental benefit over mindfulness alone or physical activity alone; and whether apparent effects vary according to intervention delivery, population, duration, or activity modality. These distinctions are important because passive and active comparators answer different clinical questions, and combining them in a single pooled estimate can obscure the incremental value of the combined approach.

We therefore conducted a systematic review and meta-analysis of randomised controlled trials evaluating combined mindfulness and physical activity interventions for stress or stress-related psychological outcomes in adult and mixed-age populations with a study-level mean or median age of at least 18 years. The objectives were to (1) estimate efficacy relative to passive or general control conditions; (2) estimate incremental benefit relative to mindfulness alone and physical activity alone in separate comparator-specific meta-analyses; and (3) explore heterogeneity according to delivery format, study-level mean age, intervention duration, participant health status, physical activity modality, and comparator type/intensity.

## 2. Methods

### 2.1. Search Strategy and Protocol

This review was reported in accordance with the Preferred Reporting Items for Systematic Reviews and Meta-Analyses 2020 statement (PRISMA 2020; Page et al., 2021). Searches were conducted during November 2025 in PubMed, Scopus, Web of Science Core Collection, EBSCOhost, ProQuest Dissertations & Theses Global, ScienceDirect, JSTOR, and Springer Nature Link. Although ProQuest was searched to identify potentially relevant records and citation leads, unpublished dissertations and theses were not eligible under the peer-reviewed publication criterion. The database-specific search strategies and search period are reported in Supplementary Table S1.

The database searches were guided by a common conceptual framework covering mindfulness- or meditation-related interventions, physical activity or structured movement, and stress or stress-related psychological outcomes. However, the breadth and combination of search terms were adapted to the indexing systems, searchable fields, and interface functions of individual databases. Where supported, controlled vocabulary (including Medical Subject Headings (MeSH) in PubMed) was combined with free-text terms, and trial-design or combined-intervention terms were incorporated when appropriate. In databases in which more complex Boolean combinations produced overly restrictive retrieval, simpler combinations were used to avoid excessive loss of potentially relevant records. Because database platforms differ in indexing and search functionality, identical search sensitivity across databases could not be assumed. The complete database-specific strategies reported in Supplementary Table S1 represent the actual searches conducted. To complement the database searches, reference lists of included studies and relevant reviews were screened, and forward citation searching was conducted in Google Scholar and Web of Science.

The review was retrospectively registered in PROSPERO on 5 August 2026 (CRD420261470380), after completion of the literature search and primary analyses. Because registration was retrospective, it did not constitute an a priori protocol.

### 2.2. Eligibility Criteria and Intervention Classification

Studies were eligible for the systematic review when they met all of the following criteria: (1) a randomised controlled trial design; (2) participants were adults, or the trial included a mixed-age sample with a reported study-level mean or median age of at least 18 years; mixed-age studies were eligible even when the reported age range extended below 18 years; (3) the intervention contained both an explicit mindfulness- or meditation-related component and a planned physical activity or movement component; (4) the comparator was a passive or general control condition, mindfulness alone, or physical activity alone; (5) at least one stress or stress-related psychological outcome was assessed using a validated measurement instrument; and (6) the report was published in English or Chinese in a peer-reviewed journal. No language filter was applied during database searching; reports published in English or Chinese were eligible for inclusion.

For this review, passive or general control conditions were defined as comparator conditions that did not constitute a structured mindfulness-alone or structured physical-activity-alone intervention. This category included inactive or minimal controls (wait-list, no-treatment, or no-intervention) as well as general comparison conditions such as educational or attention controls, usual care or usual curriculum, physical-activity advice or monitoring that did not constitute a structured physical-activity programme, and pharmacological treatment.

An intervention was classified as combined only when the original report explicitly described mindfulness- or meditation-related instruction in addition to planned physical activity or movement. Yoga, Tai Chi, and Qigong were not assumed to contain a mindfulness component solely on the basis of the activity modality. Breathing-only interventions (i.e., those without a planned movement component) were not classified as physical activity.

For this review, simultaneous delivery was defined as the provision of explicit mindfulness-related instructions during physical activity, such as directing attention to breathing, bodily sensations, movement, present-moment experience, or non-judgemental awareness. Interventions that included both separate mindfulness exercises and explicit mindfulness cueing during physical activity were classified as simultaneous. Separate delivery was defined as the provision of mindfulness and physical activity as distinguishable components before or after one another, or in different sessions, without explicit mindfulness cueing during the physical activity itself.

Availability of sufficient numerical data was not required for inclusion in the systematic review. For inclusion in the quantitative synthesis, sufficient information had to be available to calculate or reconstruct an effect estimate and its sampling variance. Studies that otherwise met the eligibility criteria but lacked sufficient quantitative information were retained in the systematic review, study-characteristics summary, narrative synthesis, and risk-of-bias assessment but were not included in the meta-analysis.

Studies were excluded from the systematic review if they used a non-randomised or non-interventional design; were case reports, single-case studies, reviews, theoretical papers, commentaries, editorials, protocols without outcome data, or duplicate reports of an overlapping sample; did not evaluate an eligible combined intervention; did not include an eligible comparator or outcome; or were unpublished or non-peer-reviewed materials.

### 2.3. Study Selection

**Figure 1:**
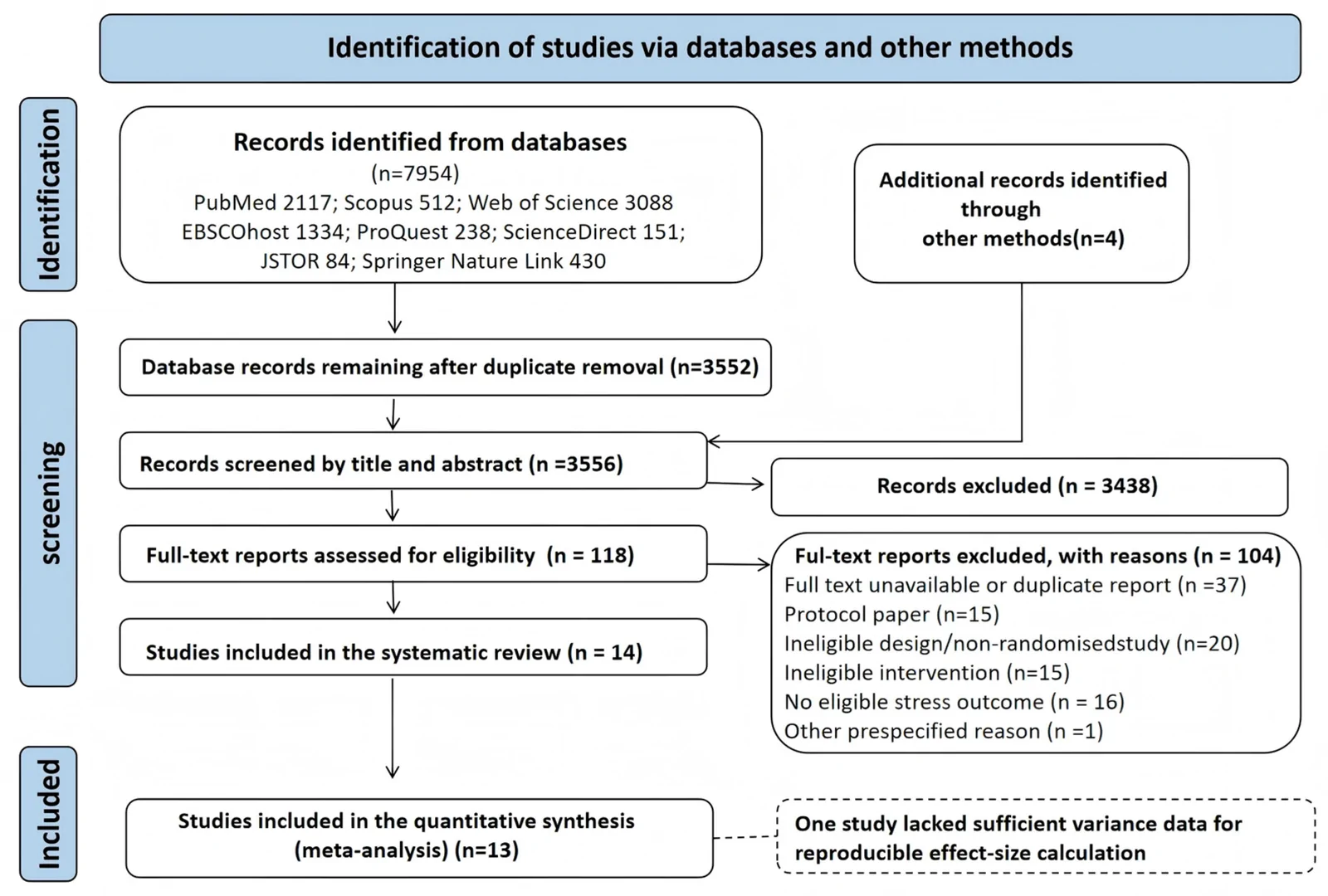
PRISMA 2020 flow diagram of the literature search and study-selection process.

All records were imported into Zotero version (7.0.32) and deduplicated. Two reviewers independently screened titles and abstracts and then independently assessed potentially eligible full-text reports. Disagreements were resolved through discussion; when consensus could not be reached, a third reviewer adjudicated. Cohen’s *κ* was calculated for title and abstract screening. The numbers of records identified, screened, excluded, assessed at full text, included in the systematic review, and included in quantitative synthesis are presented in the PRISMA flow diagram (Figure 1).

### 2.4. Data Extraction and Risk-of-Bias Assessment

Data extraction and the initial risk-of-bias assessment were performed by the first author and subsequently checked in full by a second reviewer. These procedures therefore involved one primary assessment followed by complete verification rather than fully independent duplicate assessments. Discrepancies were resolved through discussion, with final adjudication by a third author when required.

Extracted characteristics included author, year, country, participant eligibility and health status, randomised and analysed sample sizes, mean age, intervention and comparator content, delivery format, session frequency and duration, total programme duration, adherence, attrition, intervention fidelity, follow-up periods, and adverse events where reported. For each eligible outcome, we extracted the instrument name and version, construct assessed, scoring direction, assessment time points, adjusted or unadjusted status, group means, standard deviations, sample sizes, mean differences, confidence intervals, *p* values, and other information required for effect-size calculation. Measurement instruments, selected outcomes, assessment time points, and effect-size data sources are detailed in Supplementary Table S2.

Risk of bias was assessed with the Cochrane Risk of Bias tool version 1.0 (RoB 1.0; Higgins et al., 2011). The seven domains were random sequence generation, allocation concealment, blinding of participants and personnel, blinding of outcome assessment, incomplete outcome data, selective reporting, and other bias. Each domain was judged as low, unclear, or high risk of bias.

### 2.5. Outcome Selection and Effect-Size Calculation

The quantitative outcome was the between-group difference in a stress or stress-related psychological outcome at the earliest available assessment after completion of the intervention; longer-term follow-up assessments were not included in the primary analyses. To avoid outcome multiplicity, only one outcome per trial and comparator type was included. Eligible outcomes encompassed both direct measures of stress and broader stress-related psychological outcomes assessed using validated instruments. When more than one eligible outcome was reported at the selected time point, the outcome most directly representing stress was selected according to the following hierarchy: perceived stress measure, validated stress subscale, stress-related anxiety or psychological distress measure, and mental well-being measure. A sensitivity analysis was subsequently restricted to direct measures of stress.

Intervention effects were expressed as Hedges’ *g*, a small-sample bias-corrected standardised mean difference (Hedges & Olkin, 1985; Borenstein et al., 2009). For 11 trials, effect sizes were calculated from unadjusted post-intervention group means, standard deviations, and analysed sample sizes. Cohen’s *d* was calculated by dividing the difference between the combined-group and comparator-group means by the pooled post-intervention standard deviation. The small-sample correction was *J* = 1 − 3/[4(*n*_1_ + *n*_0_) − 9], and Hedges’ *g* was calculated as *Jd*. The sampling variance was calculated as *J*^2^[(*n*_1_ + *n*_0_)/(*n*_1_*n*_0_) + *d*^2^/{2(*n*_1_ + *n*_0_ − 2)}], with the standard error equal to the square root of the variance. Negative values indicated more favourable outcomes in the combined-intervention group. For positively scored outcomes, the sign was reversed to maintain this direction. Because these effects used post-intervention group data, no change-score standard deviation or assumed pre–post correlation was required.

For Shi et al. (2019), raw post-intervention standard deviations were unavailable. The adjusted change from baseline to the immediate post-intervention assessment in the combined group (−1.21) was contrasted with that in the control group (+0.62). The adjusted mean-change contrast was standardised using the pooled baseline standard deviation and corrected for small-sample bias. Because only a rounded two-sided *p* value of 0.02 was reported for the between-group contrast, its standard error was approximated from the corresponding normal-test statistic.

For Rotter et al. (2022), the reported baseline-adjusted endpoint mean difference (−1.6; 95% CI −4.8 to 1.6) was converted to a standard error using the confidence-interval width, standardised using the pooled baseline standard deviation, and corrected for small-sample bias. The reconstructed estimates from Shi et al. and Rotter et al. were entered using the generic inverse-variance approach and were excluded together in a sensitivity analysis.

Hunt et al. (2018) was retained in the systematic review but was not included in the quantitative synthesis because exact group-level variance data were not reported and the error bars in the published figure were not defined sufficiently to permit a reproducible effect-size calculation. No unreported means or standard deviations were imputed.

In multi-arm trials, a study could contribute one effect estimate to more than one clinically distinct comparator-specific meta-analysis, but it contributed no more than one estimate to any single model. Shared intervention groups were therefore not duplicated within a pooled estimate. Because the three comparator types were analysed in separate models and no cross-comparator overall estimate was calculated, within-study dependence across comparator-specific models did not affect the variance of any single pooled estimate.

### 2.6. Meta-Analysis, Subgroup, Sensitivity, and Small-Study-Effect Analyses

All statistical analyses were conducted using Stata version 18.0. Three separate primary meta-analyses were performed: combined intervention versus passive or general control, versus mindfulness alone, and versus physical activity alone. These comparisons were not combined because they address different clinical questions. Random-effects models used restricted maximum-likelihood estimation of the between-study variance and Knapp–Hartung standard errors (Viechtbauer, 2005; IntHout et al., 2014). Results are reported as pooled Hedges’ *g* values with 95% confidence intervals. Heterogeneity was evaluated using Cochran’s *Q*, *I*^2^ (Higgins et al., 2003), and *τ*^2^. A 95% prediction interval was calculated when at least three studies were available and was interpreted cautiously in small meta-analyses (IntHout et al., 2016). Statistical tests were two-sided, with *p* < 0.05 considered statistically significant.

Exploratory subgroup analyses were restricted to the passive or general control comparison. The moderators were delivery format (simultaneous versus separate), study-level mean age (<40 versus ≥40 years), intervention duration (<8 versus ≥8 weeks), participant health status (healthy/nonclinical, subclinical/high-stress, or clinical condition), broad physical activity modality (mind–body movement/yoga/Tai Chi, walking/general exercise or physical activity, or stretching/resistance training), and comparator type/intensity (inactive/minimal versus active/general control conditions). Inactive/minimal controls comprised wait-list, no-treatment, or no-intervention conditions, whereas active/general controls comprised educational or attention conditions, usual care or usual curriculum, physical-activity advice or monitoring, and pharmacological treatment. The 40-year threshold represented an approximate median split of study-level mean ages and did not classify individual participants. The 8-week threshold was selected because 8 weeks was the modal programme duration. Formal between-subgroup *Q* tests were used; moderation was inferred only from the between-subgroup test and not from differences in the statistical significance of subgroup-specific pooled estimates. Owing to the small number of studies within several categories, all subgroup analyses were considered exploratory.

Sensitivity and influence analyses for the passive or general control comparison included exclusion of the reconstructed Shi and Rotter estimates; exclusion of Wang et al. (2024); leave-one-study-out analysis in which the complete trial was removed at each iteration; DerSimonian–Laird random-effects (DerSimonian & Laird, 1986) and common-effect inverse-variance models; restriction to direct stress measures. The Galbraith plot was used as an additional graphical assessment of outlying and influential estimates (Galbraith, 1988). The DerSimonian–Laird and common-effect analyses were treated as sensitivity analyses and were not used to override the primary REML model.

Small-study effects were examined only for the passive or general control comparison, which included 12 studies. Standard and contour-enhanced funnel plots were inspected, and Egger’s regression test was performed (Egger et al., 1997; Sterne et al., 2000). A non-significant Egger test was interpreted as failure to detect statistically significant small-study effects rather than proof of the absence of publication bias. Trim-and-fill was performed as an exploratory analysis (Duval & Tweedie, 2000). These analyses were not performed for the mindfulness-alone or physical-activity-alone comparisons because too few studies were available.

### 2.7. Assessment of Certainty of Evidence

The certainty of evidence for each of the three comparator-specific primary analyses was assessed using the GRADE approach (Guyatt et al., 2011). Because all included studies were randomised controlled trials, certainty for each comparison started at high and could be downgraded for risk of bias, inconsistency, indirectness, imprecision, and publication bias. This assessment was conducted during manuscript revision in response to reviewer comments and was not part of the retrospectively registered review plan. The initial GRADE assessment was performed by one reviewer and checked in full by a second reviewer, with disagreements resolved through discussion. The resulting certainty ratings are presented in Supplementary Table S3.

## 3. Results

### 3.1. Study Selection and Characteristics of Included Trials

The database searches identified 7954 records, and four additional records were identified through reference-list screening and citation searching. After duplicate removal, 3552 database records remained. The four additional unique records were then added, resulting in 3556 records for title and abstract screening. Agreement between the two reviewers during title and abstract screening was excellent (Cohen’s *κ* = 0.82). Of the screened records, 3438 were excluded, and 118 full-text reports were assessed for eligibility. A total of 104 reports were excluded for prespecified reasons (Figure 1).

A total of 14 randomised controlled trials, involving 872 randomised participants, met the eligibility criteria and were included in the systematic review. Of these, 13 trials contributed data to at least one comparator-specific meta-analysis. Hunt et al. (2018) was not included in the quantitative synthesis because the published report did not provide sufficiently defined variance information to permit a reproducible effect-size calculation. The study remained included in the systematic review, study-characteristics summary, narrative synthesis, and risk-of-bias assessment.

The included trials evaluated heterogeneous combinations of mindfulness- or meditation-related practices and physical activity across healthy or nonclinical participants, subclinical or high-stress populations, and participants with clinical conditions. Intervention durations ranged from 4 to 12 weeks. Stress or stress-related outcomes were assessed using versions of the Perceived Stress Scale, the Perceived Stress Questionnaire, the stress subscale of the DASS-21, the State-Trait Anxiety Inventory, the Brief Symptom Rating Scale, and the Warwick–Edinburgh Mental Well-Being Scale. Detailed information on the instrument and assessment time point used for each study is provided in Supplementary Table S2.

Twelve trials contributed to the comparison with a passive or general control condition, two trials contributed to the comparison with mindfulness alone, and three trials contributed to the comparison with physical activity alone. Because some multi-arm trials contributed to more than one comparator-specific analysis, these numbers are not additive. Each trial contributed no more than one effect estimate to any single comparator-specific meta-analysis. The characteristics of the included studies are summarised in Table 1.

### 3.2. Risk-of-Bias Assessment

**Figure 2:**
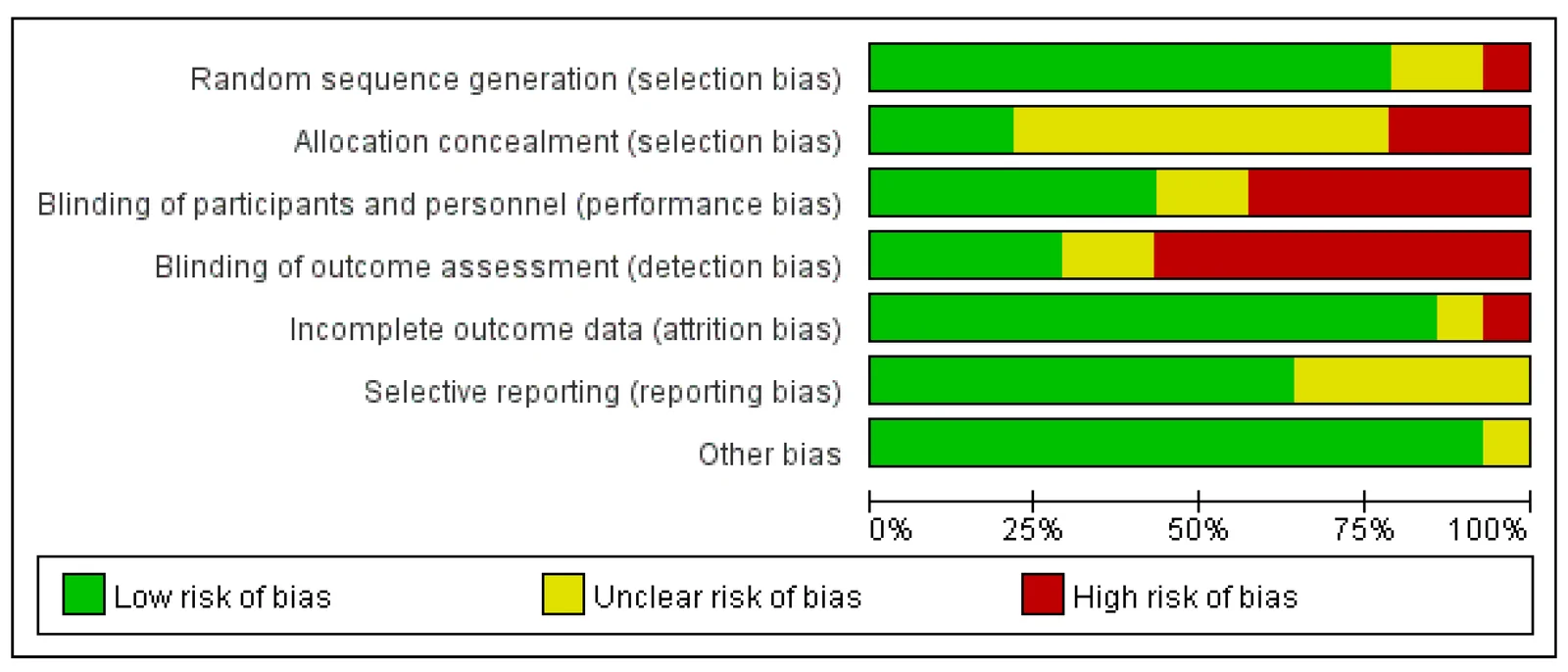
Summary of risk-of-bias judgements across the 14 included trials according to the Cochrane RoB 1.0 tool.

**Figure 3:**
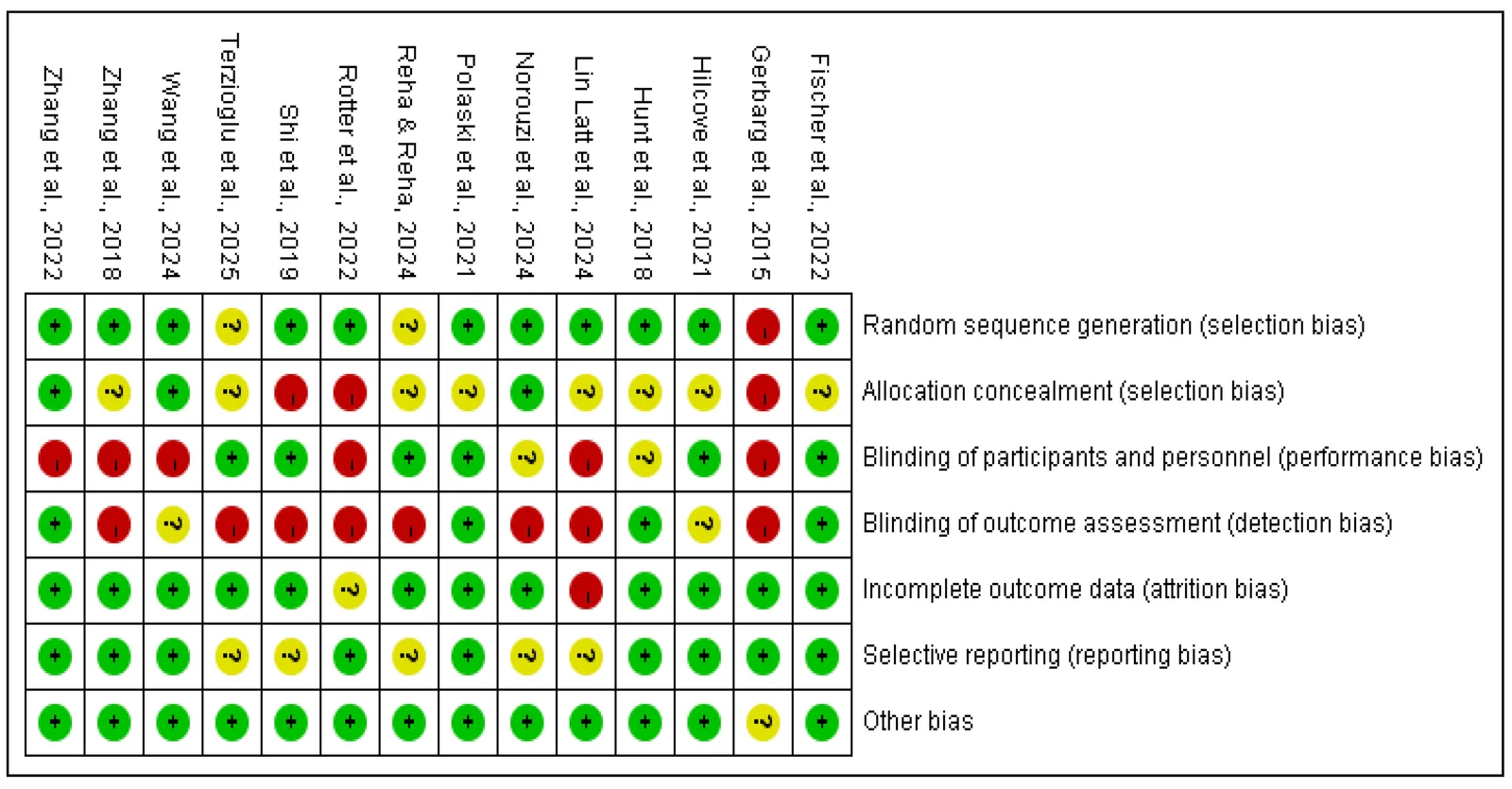
Study-level risk-of-bias judgements according to the Cochrane RoB 1.0 tool. Green (+), low risk; yellow (?), unclear risk; red (−), high risk (J.-Y. Zhang et al., 2022; J. Zhang et al., 2018; Wang et al., 2024; Terzioğlu & Çakır-Çelebi, 2025; Shi et al., 2019; Rotter et al., 2022; Reha & Reha, 2024; Polaski et al., 2021; Norouzi et al., 2024; Lin Latt et al., 2024; Hunt et al., 2018; Hilcove et al., 2021; Gerbarg et al., 2015; Fischer et al., 2022).

Risk of bias was assessed using the Cochrane Risk of Bias tool version 1.0 (RoB 1.0). Random sequence generation was judged to be at low risk in 11 of the 14 trials, unclear in two trials, and high risk in one trial. Allocation concealment was judged to be at low risk in three trials, unclear in eight trials, and high risk in three trials. High-risk judgements were most common for blinding of outcome assessment (7/14 trials) and blinding of participants and personnel (6/14 trials), reflecting the practical difficulty of blinding behavioural interventions and the frequent use of self-reported outcomes. Incomplete outcome data was judged to be at low risk in 12 trials, unclear in one trial, and high risk in one trial. Selective reporting was judged to be at low risk in nine trials and unclear in five trials, whereas other bias was judged to be at low risk in 13 trials and unclear in one trial. The domain-level distribution of judgements and the study-level assessments are presented in Figure 2 and Figure 3, respectively.

### 3.3. Primary Outcome Analysis

Negative Hedges’ *g* values indicated more favourable outcomes in the combined-intervention group. The three comparator types were analysed separately, and no pooled estimate was calculated across comparator types.

**Figure 4:**
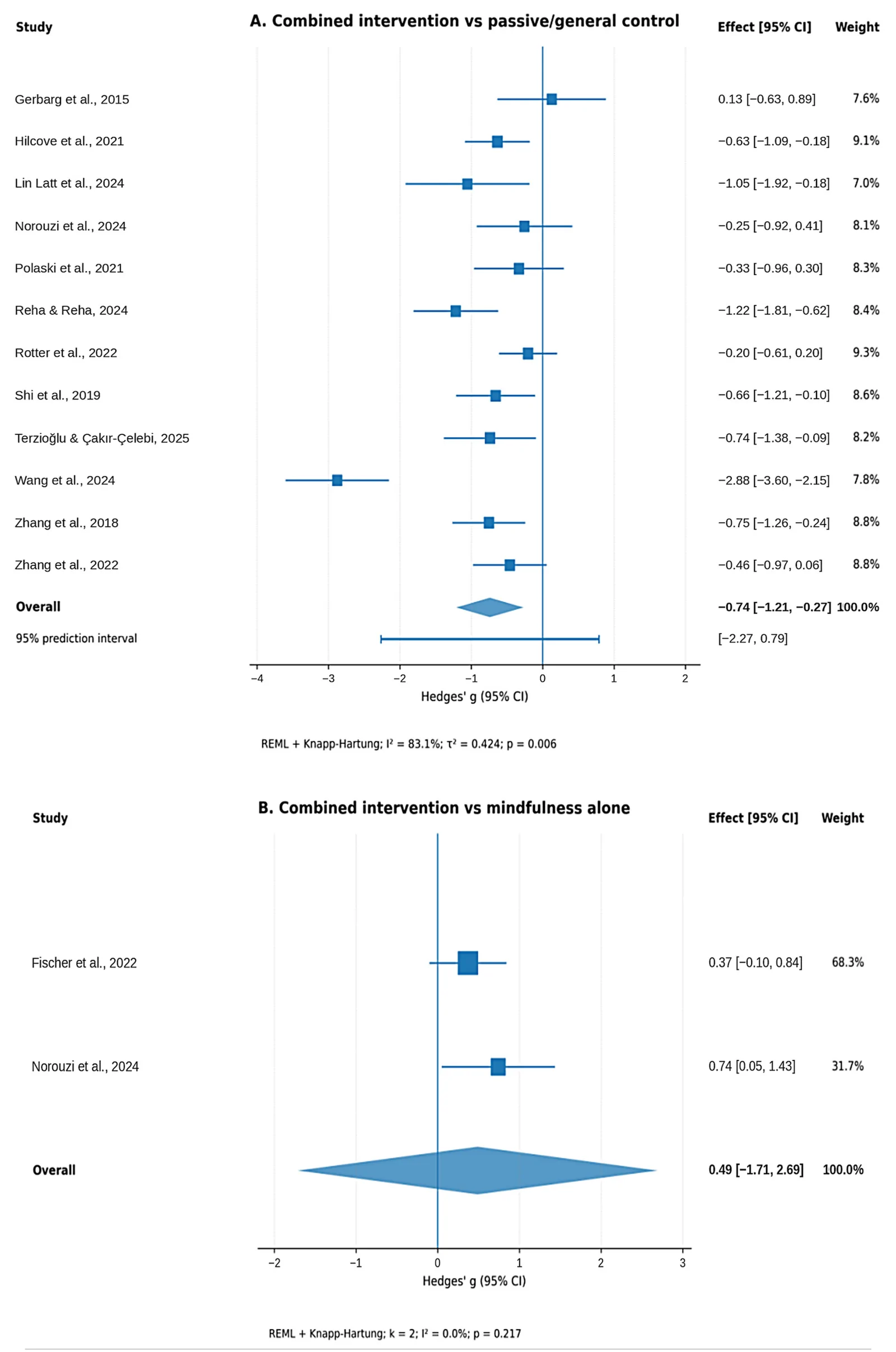

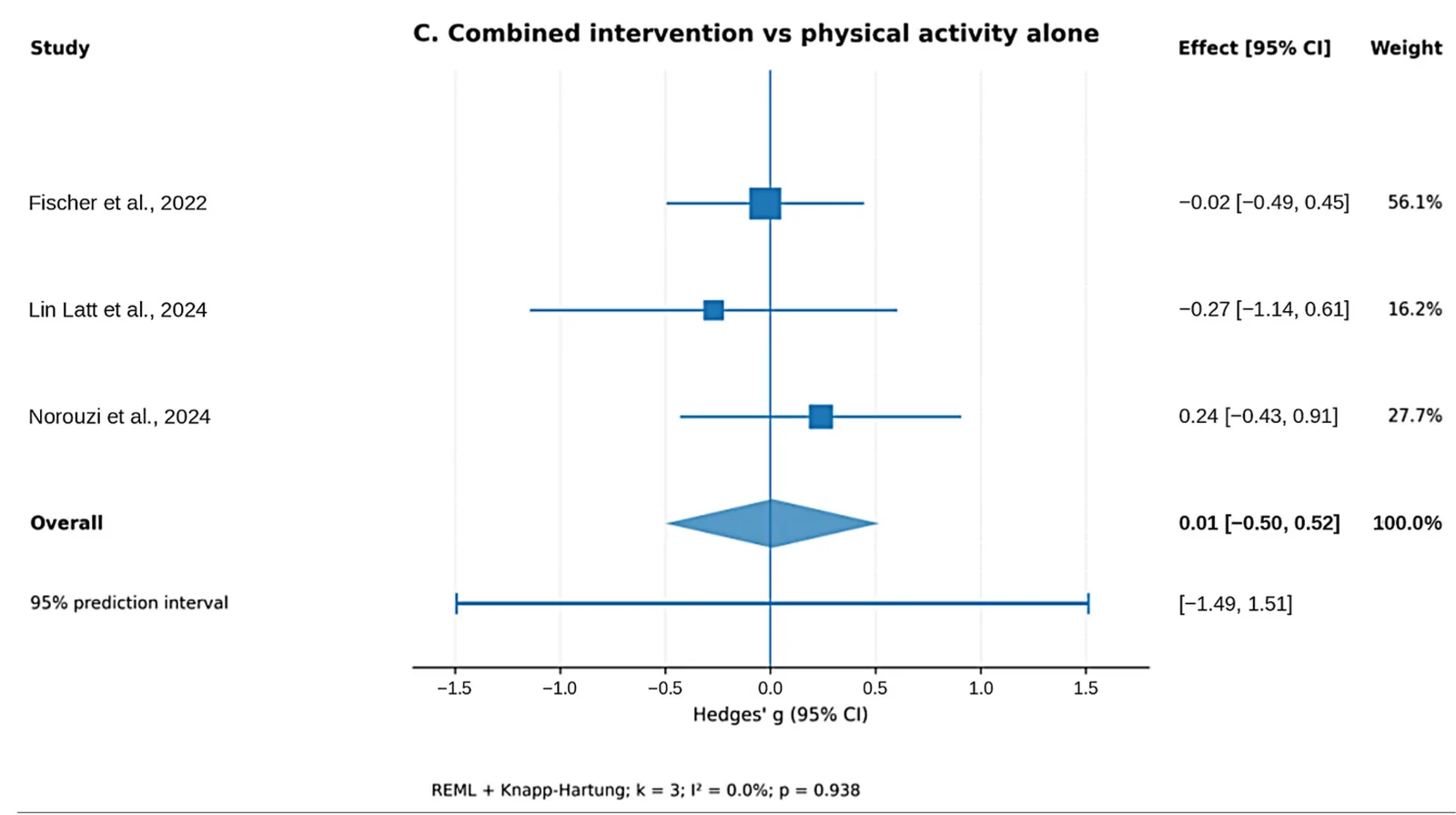
Negative values favour the combined intervention. Random-effects models used restricted maximum-likelihood estimation with Knapp–Hartung adjustment. (Gerbarg et al., 2015; Hilcove et al., 2021; Lin Latt et al., 2024; Norouzi et al., 2024; Polaski et al., 2021; Reha & Reha, 2024; Rotter et al., 2022; Shi et al., 2019; Terzioğlu & Çakır-Çelebi, 2025; Wang et al., 2024; J. Zhang et al., 2018; J.-Y. Zhang et al., 2022; Fischer et al., 2022). Squares represent study-specific effect estimates, with square size proportional to study weight; horizontal lines represent 95% confidence intervals; diamonds represent pooled effect estimates; where shown, the horizontal line labelled “95% prediction interval” represents the 95% prediction interval.

For combined interventions versus passive or general control conditions, 12 studies were included. The REML random-effects model with Knapp–Hartung adjustment showed a statistically significant average effect favouring the combined intervention (Hedges’ *g* = −0.74, 95% CI −1.21 to −0.27; *p* = 0.006). Heterogeneity was substantial (*τ*^2^ = 0.424; *I*^2^ = 83.1%; *Q*(11) = 52.39, *p* < 0.001). The 95% prediction interval ranged from −2.27 to 0.79, indicating that the true effect in a new setting could vary considerably and may include little or no benefit (Figure 4A).

Two studies compared the combined intervention with mindfulness alone. The pooled effect was not statistically significant and was highly imprecise (Hedges’ *g* = 0.49, 95% CI −1.71 to 2.69; *p* = 0.217; *I*^2^ = 0.0%; *τ*^2^ = 0.000) (Figure 4B). Given the small number of studies and the wide confidence interval, this analysis did not provide sufficient evidence that the combined intervention was superior to mindfulness alone and should not be interpreted as evidence of equivalence.

Three studies compared the combined intervention with physical activity alone. The pooled effect was close to the null and was not statistically significant (Hedges’ *g* = 0.01, 95% CI −0.50 to 0.52; *p* = 0.938; *I*^2^ = 0.0%; *τ*^2^ = 0.000). The 95% prediction interval was −1.49 to 1.51 and was highly uncertain because only three studies contributed to this comparison (Figure 4C). This analysis did not demonstrate statistically significant superiority of the combined intervention over physical activity alone.

### 3.4. Subgroup Analyses

Exploratory subgroup analyses were conducted only for the comparison between the combined intervention and passive or general control conditions. Formal between-subgroup tests were used to evaluate subgroup differences.

By delivery format, the pooled effect was −0.46 (95% CI −0.77 to −0.16; *k* = 6) for simultaneous delivery and −1.07 (95% CI −2.08 to −0.07; *k* = 6) for separate delivery. The formal between-subgroup difference was not statistically significant (*Q_b_* = 2.23, *p* = 0.135). Thus, the analysis did not establish delivery format as a statistically significant moderator.

By study-level mean age, the pooled effect was −0.72 (95% CI −1.11 to −0.33; *k* = 6) in studies with a mean age below 40 years and −0.77 (95% CI −1.87 to 0.33; *k* = 6) in studies with a mean age of 40 years or older. The between-subgroup difference was not statistically significant (*Q_b_* = 0.01, *p* = 0.905).

By intervention duration, the pooled effect was −0.65 (95% CI −1.03 to −0.28; *k* = 7) for interventions lasting less than 8 weeks and −0.89 (95% CI −2.26 to 0.48; *k* = 5) for interventions lasting 8 weeks or longer. The between-subgroup difference was not statistically significant (*Q_b_* = 0.22, *p* = 0.641).

Additional exploratory analyses examined participant health status and broad physical-activity modality. The pooled effects were −0.71 (95% CI −0.94 to −0.47; *k* = 4) in healthy or nonclinical samples, −0.96 (95% CI −3.88 to 1.97; *k* = 2) in subclinical or high-stress samples, and −0.66 (95% CI −1.80 to 0.48; *k* = 6) in clinical samples. The between-subgroup difference was not statistically significant (*Q_b_* = 0.91, *p* = 0.635). By physical-activity modality, pooled effects were −0.63 (95% CI −1.17 to −0.08; *k* = 5) for mind–body movement, yoga, or tai chi; −0.85 (95% CI −2.25 to 0.55; *k* = 5) for walking, general exercise, or general physical activity; and −0.85 (95% CI −2.77 to 1.07; *k* = 2) for stretching or resistance training. The between-subgroup difference was not statistically significant (*Q_b_* = 0.58, *p* = 0.747). By comparator type/intensity, the pooled effect was −0.65 (95% CI −1.04 to −0.26; *k* = 6; *I*^2^ = 47.3%) for inactive/minimal controls and −0.79 (95% CI −1.89 to 0.32; *k* = 6; *I*^2^ = 90.5%) for active/general controls. The formal between-subgroup difference was not statistically significant (*Q_b_* = 0.09, *p* = 0.765). None of the formal subgroup tests identified a statistically significant moderator (Table 2; Supplementary Figures S1–S6).

### 3.5. Sensitivity, Influence, and Small-Study-Effect Analyses

The primary control-comparison result was robust to several sensitivity analyses. After excluding the reconstructed effect estimates from Shi et al. (2019) and Rotter et al. (2022), the pooled effect remained statistically significant (*k* = 10; Hedges’ *g* = −0.81, 95% CI −1.39 to −0.23; *p* = 0.011; *I*^2^ = 84.2%; *τ*^2^ = 0.515; 95% prediction interval −2.57 to 0.95).

Wang et al. (2024) contributed the most extreme effect estimate. Excluding this study reduced the pooled effect to −0.55 (95% CI −0.79 to −0.31; *p* < 0.001) and markedly reduced heterogeneity (*I*^2^ = 27.9%; *τ*^2^ = 0.032; 95% prediction interval −1.02 to −0.08). This finding indicates that Wang et al. was an important contributor to heterogeneity, although its exclusion did not change the direction or statistical significance of the pooled result.

In the leave-one-study-out analysis, pooled estimates ranged from −0.81 to −0.55, and all 95% confidence intervals excluded zero. The DerSimonian–Laird random-effects model produced a similar pooled estimate (Hedges’ *g* = −0.74, 95% CI −1.10 to −0.37; *I*^2^ = 79.0%; *τ*^2^ = 0.325), while the common-effect inverse-variance model produced an estimate of −0.66 (95% CI −0.83 to −0.50). These alternative models were treated as sensitivity analyses rather than as evidence that heterogeneity was unimportant.

When the analysis was restricted to nine studies using direct measures of stress, the pooled effect remained statistically significant (Hedges’ *g* = −0.56, 95% CI −0.85 to −0.26; *p* = 0.003), and heterogeneity was reduced (*I*^2^ = 39.0%; *τ*^2^ = 0.051). The corresponding 95% prediction interval was −1.17 to 0.06.

Visual inspection of the standard and contour-enhanced funnel plots did not indicate a clear pattern of small-study effects, although interpretation was limited by the number of studies and between-study heterogeneity. Egger’s regression test did not indicate statistically significant small-study effects (*β* = −3.16, SE = 3.26, *z* = −0.97, *p* = 0.333). Exploratory trim-and-fill analysis imputed no missing studies and did not change the pooled estimate. The Galbraith plot and influence analyses were consistent with Wang et al. (2024) being the principal outlying estimate (Supplementary Figures S7–S12).

### 3.6. Certainty of Evidence

Using the GRADE approach, the certainty of evidence was rated low for the comparison with passive or general control conditions, very low for the comparison with mindfulness alone, and low for the comparison with physical activity alone. The passive/general-control comparison was downgraded for risk of bias and serious inconsistency. The mindfulness-alone comparison was downgraded for risk of bias and very serious imprecision, whereas the physical-activity-alone comparison was downgraded for risk of bias and serious imprecision. No downgrade for indirectness or publication bias was applied. Detailed judgements and reasons for downgrading are provided in Supplementary Table S3.

## 4. Discussion

This systematic review included 14 randomised controlled trials, 13 of which contributed to at least one comparator-specific meta-analysis. The revised analyses yielded three clinically distinct findings. First, combined mindfulness and physical activity interventions were associated with lower stress or stress-related outcome scores than passive or general control conditions (Hedges’ *g* = −0.74, 95% CI −1.21 to −0.27). However, heterogeneity was substantial, and the 95% prediction interval (−2.27 to 0.79) crossed the null, indicating that the average pooled effect should not be interpreted as a uniform benefit across populations, interventions, and settings. Second, the available evidence did not demonstrate statistically significant superiority over mindfulness alone or physical activity alone. These active-comparator analyses were based on only two and three trials, respectively, and their confidence intervals were wide. They therefore indicate uncertainty about incremental benefit rather than equivalence, absence of synergy, or proof that the combined approach is unnecessary. Third, none of the formal subgroup tests identified a statistically significant moderator. The influence and sensitivity analyses supported the direction of the primary control-comparison result, while also showing that the estimate from Wang et al. (2024) was an important contributor to between-study heterogeneity.

### 4.1. Effects Relative to Passive or General Control Conditions

The significant average effect relative to passive or general control conditions is consistent with the established stress-management rationale for both intervention components. Mindfulness-based practices may influence stress through attentional regulation, reduced rumination, greater acceptance of difficult internal experiences, and improved emotion regulation, whereas physical activity may affect stress through behavioural activation, improved sleep and affect, and physiological pathways involving stress reactivity and neuroendocrine regulation (Bohlmeijer et al., 2010; Jain et al., 2007; Tang et al., 2015; Creswell et al., 2019). The present findings support the potential value of programmes that combine these components when the relevant alternative is wait-list, usual care, or another minimal-intervention condition.

This interpretation nevertheless requires caution. The control-comparison studies included markedly different populations, mindfulness practices, physical activity components, delivery procedures, and outcome instruments. Moreover, the prediction interval included the possibility of little or no benefit in a new setting. Thus, the pooled estimate represents an average across a heterogeneous group of interventions rather than the expected effect of a single standardised programme. The sensitivity analysis restricted to direct measures of stress produced a smaller but still statistically significant pooled effect (Hedges’ *g* = −0.56) and reduced heterogeneity, suggesting that part of the variability in the primary analysis arose from combining perceived stress with broader stress-related constructs. Accordingly, the primary pooled estimate should be interpreted as reflecting a broader stress-related psychological outcome domain, whereas the sensitivity analysis restricted to direct measures of stress offers a more construct-specific estimate focused on stress itself.

### 4.2. Incremental Benefit over Single-Component Interventions

The active-comparator analyses address a different question from the comparison with passive or general controls. They assess whether a combined mindfulness and physical activity intervention provides additional benefit relative to mindfulness alone or physical activity alone. The pooled analyses did not demonstrate statistically significant superiority over either mindfulness alone or physical activity alone. However, the mindfulness comparison included only two trials, and the physical activity comparison included only three. The resulting estimates were imprecise and compatible with effects favouring either the combined or the single-component intervention.

Accordingly, the current evidence does not justify concluding that the components are equivalent, that synergy is absent, or that an additive model has been established. Demonstrating synergy requires designs and analyses capable of estimating component effects and their interaction, ideally through adequately powered factorial or dismantling trials. Differences among the mindfulness components are also important: structured MBSR, brief meditation, mindful self-hypnosis, mindful walking, and mindfulness-based yoga or Tai Chi should not be treated as interchangeable. The same applies to walking, resistance training, stretching, yoga, Tai Chi, and mixed exercise programmes. Future head-to-head studies should report the dose, fidelity, adherence, and quality of each component so that a null incremental effect can be distinguished from an under-dosed or poorly implemented combination.

### 4.3. Heterogeneity and Exploratory Moderators

None of the formal between-subgroup tests was statistically significant. In particular, the difference between simultaneous and separate delivery was not significant (*p* = 0.135). The data therefore do not establish delivery format as an effect modifier and do not support recommending separate delivery as more effective. Similarly, the analyses did not demonstrate statistically significant differences by study-level mean age, intervention duration, participant health status, broad physical activity modality, or comparator type/intensity. The exploratory comparison between inactive/minimal and active/general controls was likewise not statistically significant (*Q_b_* = 0.09, *p* = 0.765). However, substantial residual heterogeneity remained within the active/general-control subgroup (*I*^2^ = 90.5%), indicating that this broad category continued to encompass clinically heterogeneous comparison conditions. Recommendations for programmes lasting at least eight weeks are therefore not supported by these subgroup results.

These analyses should be interpreted as exploratory because several categories contained few studies, subgroup definitions were broad, and substantial residual heterogeneity remained within some categories. The age analysis was based on study-level mean age and cannot determine whether individual participant age modified treatment effects. The health-status analysis combined distinct clinical conditions, and the physical activity categories grouped interventions with potentially different mechanisms and doses. A non-significant subgroup test may therefore reflect either a true lack of moderation or limited statistical power and imprecise classification. Individual-participant-data meta-analysis or larger sets of consistently reported trials would be better suited to evaluating these questions.

### 4.4. Robustness, Influence, and Risk of Bias

The principal control-comparison result remained statistically significant after excluding the reconstructed effect estimates from Shi et al. (2019) and Rotter et al. (2022), and all estimates in the leave-one-study-out analysis remained on the beneficial side of the null. These findings reduce concern that the overall direction of the result depended on a single study or on the two reconstructed estimates. Results were also similar under DerSimonian–Laird and common-effect models, although such agreement does not remove the clinical and statistical heterogeneity captured by the primary REML model and prediction interval.

Wang et al. (2024) produced the most extreme individual estimate. Excluding this study reduced the pooled effect from −0.74 to −0.55 and reduced *I*^2^ from 83.1% to 27.9%, while the pooled result remained statistically significant. This supports describing Wang et al. as an important source of heterogeneity. It does not, by itself, demonstrate that clinical population status or any other study characteristic is a confirmed effect modifier, particularly because the formal health-status subgroup test was not significant.

The risk-of-bias assessment also limits confidence in the magnitude of the pooled effect. Blinding of participants and personnel is generally impractical in behavioural interventions, and several studies had high or unclear risk in allocation concealment and outcome-assessment blinding. Many outcomes were self-reported, which may increase susceptibility to expectancy and reporting effects. Funnel-plot inspection, Egger’s test, and trim-and-fill did not indicate statistically significant small-study effects, but these analyses had limited power and cannot establish the absence of publication or selective-reporting bias.

### 4.5. Implications for Practice and Research

For practice, the findings suggest that combined mindfulness and physical activity programmes may be a reasonable option for reducing stress or stress-related outcomes when compared with minimal intervention or usual care. The evidence does not show that a combined programme should routinely be preferred to a well-delivered single-component intervention. Decisions should therefore consider participant preference, accessibility, safety, cost, time burden, and whether each component addresses an independently relevant clinical or behavioural goal.

Future trials should use adequately powered factorial, dismantling, or multi-arm designs; prespecify the primary stress outcome and assessment time point; report complete data for effect-size calculation; and document component dose, adherence, fidelity, co-interventions, and adverse events. Longer follow-up is needed to determine durability. Consistent reporting would also allow more informative analyses by population, mindfulness approach, physical activity modality, and delivery format, rather than relying on broad study-level categories.

## 5. Strengths and Limitations

This review has several strengths, including the restriction to randomised controlled trials, separate analyses by comparator type, use of REML random-effects models with Knapp–Hartung adjustment, reporting of prediction intervals, and multiple sensitivity and influence analyses. Several limitations should also be acknowledged. The active-comparator evidence base was relatively small, and substantial heterogeneity was observed in the passive/general-control comparison, reflecting variation in populations, intervention components, and outcome measures. The passive/general-control category also encompassed heterogeneous comparator conditions. Although the exploratory comparator type/intensity subgroup test was not statistically significant, substantial heterogeneity remained within the active/general-control subgroup, and the small number of studies limited power to detect moderation. The primary analysis included both direct measures of stress and broader stress-related psychological constructs; although related, these constructs are not equivalent and may have contributed to heterogeneity in the pooled estimate. Some effect estimates required reconstruction from incompletely reported data, and one eligible study could not contribute to the quantitative synthesis because variance information was insufficient. Risk-of-bias concerns and the frequent use of self-reported outcomes may also have influenced the estimated effects. In addition, subgroup analyses were exploratory and based on study-level characteristics, and one included study contained a mixed-age sample extending below 18 years. Finally, long-term follow-up was limited, and the modest number of studies constrained formal assessment of small-study effects. These considerations should be taken into account when interpreting the pooled estimates, particularly for the active-comparator analyses.

## 6. Conclusions

Combined mindfulness and physical activity interventions were associated with lower stress or stress-related outcomes than passive or general control conditions, although substantial heterogeneity and a wide prediction interval suggest that effects may vary across settings. Evidence remains insufficient to determine whether combined programmes provide additional benefit over mindfulness alone or physical activity alone, and no examined subgroup factor was identified as a statistically significant moderator. These findings support combined programmes as a potentially useful stress-management option, while further adequately powered component-comparison trials with standardised reporting, fidelity assessment, and longer-term follow-up are needed to clarify incremental benefit and the conditions under which these interventions are most effective.

## Figures and Tables

**Table 1:** Characteristics of included studies.

Study (Year), Country	Population and Age, Mean (SD), Years	Sample Size and Attrition	Combined Intervention and Dose	Comparator (s)	Delivery	Outcome Used and Reporting Details
Gerbarg et al. (2015), USA	Inflammatory bowel disease; BBMW 49.3 (14.2), education 58.6 (16.2)	29 randomised (15/14); 25 analysed for PSQ at 6 weeks (13/12); outcome-specific missing data; one dropout/group by 26 weeks	Breath–Body–Mind Workshop: 9 h, 2-day workshop; coherent breathing, Qigong movements coordinated with breathing, and Open Focus meditation; weekly 90 min follow-up for 6 weeks	Time-matched IBD educational seminar	Integrated/mixed delivery	PSQ at 6 weeks; 26-week follow-up; home-practice and attendance records incomplete; fidelity NR
Polaski et al. (2021), USA	Chronic low back pain; intervention 36.3 (14.1), audiobook control 38.7 (16.8)	52 randomised; 38 analysed (18/20); 14 lost to follow-up (26.9%)	Guided mindfulness meditation (12–17 min) immediately followed by moderate-intensity walking (30 min), 5 days/week for 4 weeks	Audiobook active control	Sequential: mindfulness immediately before walking	STAI at 4 weeks; no longer-term follow-up; ≥80% session completion required for analysis
Wang et al. (2024), China	Stage II–IV NSCLC; intervention 54.4 (8.1), usual care 54.4 (10.0)	60 randomised and analysed (30/30); attrition NR	MBSR plus prescribed exercise: weekly 1–1.5 h group sessions for 8 weeks and daily 35–45 min assignments	Routine health education and psychological nursing	Separate components within one programme	BSRS-5; earliest eligible post-intervention assessment; adherence/fidelity/adverse events NR
Terzioğlu and Çakır-Çelebi (2025), Türkiye	Health-sciences students; overall mean age 19.2 (SD NR)	44 randomised; 38 analysed (19/19); 6 withdrew before study participation (13.6%)	Six weekly 90 min online mindfulness/self-compassion sessions; 30 min static and dynamic stretching at the end of sessions 2–5	No-treatment control	Sequential within sessions	WEMWBS at post-test; follow-up assessment reported; programme evaluation collected; fidelity/adverse events NR
Reha and Reha (2024), Türkiye	Women who had experienced domestic violence; overall 36.1 (8.3)	59 randomised (30/29); 51 analysed (27/24); 8 withdrew or missed post-test (13.6%)	Five weekly 90 min online yoga- and mindfulness-based psychoeducation sessions plus daily 10–20 min yoga or meditation homework	Wait-list control	Blended but distinguishable components	DASS-21 stress at 5 weeks; no longer-term follow-up; no emergency-care use reported
Shi et al. (2019), USA	Adults with inadequate physical activity; intervention 52.7 (15.3), control 46.5 (12.4)	38 randomised (17/21); 37 analysed (16/21); one intervention participant withdrew (2.6%)	Four weekly 1 h mindful-walking sessions with attention to breathing, steps, and present-moment experience	Physical-activity advice plus Fitbit-HR (Fitbit, Inc., San Francisco, CA, USA)	Integrated mindfulness during walking	PSS immediately post-intervention; 1-month follow-up; attendance/adverse events NR
J. Zhang et al. (2018), China	College students with subthreshold depression; age range 16–19 years; overall mean 18.4 (2.0)	64 randomised (32/32); 62 analysed (32/30); 2 lost to follow-up (3.1%)	Mindfulness-based Tai Chi Chuan, 90 min twice weekly for 8 weeks	Usual physical-education curriculum	Integrated mindfulness during Tai Chi	Chinese PSS after 8 weeks; no longer-term follow-up; blinded outcome assessment reported
J.-Y. Zhang et al. (2022), China	Breast-cancer survivors; MTCC 47.8 (5.1), wait-list 47.2 (7.7)	59 enrolled/randomised; 58 analysed at immediate post-test (29/29); one participant excluded before 1-year follow-up	Nurse-led mindfulness-based Tai Chi Chuan, twice weekly for 8 weeks; 1 h sessions	Wait-list control	Integrated mindfulness during Tai Chi	PSS immediately post-intervention; 1-year follow-up; all MTCC participants reportedly completed 16 sessions
Hilcove et al. (2021), USA	Nurses and health-care professionals; intervention 42.4, control 42.5 (group SDs NR)	80 randomised (41/39); 78 analysed (41/37); 2 control participants withdrew (2.5%)	Mindfulness-based Hatha/Raja yoga weekly for 6 weeks; breath awareness, body scan, mindful attention during poses, and home practice 3–5 times/week	No-intervention control	Integrated mindfulness during yoga	PSS after 6 weeks; no longer-term follow-up; attendance and home-practice logs collected
Fischer et al. (2022), Germany	Adults with elevated distress; integrative yoga 46.0 (11.5), Iyengar yoga 48.4 (10.6), mindfulness 45.7 (12.5)	102 randomised and included in ITT (35/33/34); 84 completed; 18 lost to follow-up (17.6%)	Twelve-week integrative yoga combining postures, mindfulness, and ethical/philosophical elements	Iyengar yoga; mindfulness training alone	Integrated mindfulness during yoga	PSS at 12 weeks; 24-week follow-up; mean class attendance reported; ITT analysis
Norouzi et al. (2024), Iran	Outpatients with major depressive disorder; combined 32.8 (8.2), PA 33.1 (6.6), MBSR 36.7 (6.1), control 38.7 (10.3)	67 randomised and analysed (16/17/17/17); attrition NR	MBSR plus supervised physical activity for 8 weeks as augmentation to sertraline	Physical activity alone; MBSR alone; sertraline control	Separate MBSR and PA components	PSS at 8 weeks; 4-week follow-up; adherence/fidelity/adverse events NR
Lin Latt et al. (2024), USA	Female college students; overall 21.0 (2.3)	44 randomised (16/14/14); 30 analysed (11/8/11); 14 dropped out or were excluded (31.8%)	Five-week resistance-training programme with approximately 5 min of mindful self-hypnosis immediately before and after each RT session	Resistance training alone; wait-list control	Sequential: mindfulness before and after RT	PSS at 5 weeks; no longer-term follow-up; training frequency and satisfaction reported
Hunt et al. (2018), USA	Undergraduate students; overall 19.3 (1.2)	119 randomised across two phases (22 combined/23 mindfulness/24 yoga/26 active placebo/24 no treatment); 110 completed the post-treatment stress challenge	Four-session combined mindfulness training/meditation and yoga programme	Mindfulness alone; yoga alone; active placebo study break; no treatment	Separate mindfulness and yoga components	STAI and PANAS; weekly and post-treatment assessments; quantitative meta-analysis excluded because variance information was not reproducible
Rotter et al. (2022), Germany	Chronic low back pain; mindful walking 52.5 (8.6), control 54.8 (7.5)	55 randomised (29/26); 48 available-case PSS analyses at 8 weeks; 8 withdrew before the first intervention	Eight weekly 50–60 min mindful-walking sessions with explicit mindfulness instructions during walking	No-intervention control	Integrated mindfulness during walking	PSS-14 at 8 weeks; 12-week follow-up; session attendance and adverse events reported

Note. Randomised n denotes the number allocated to study arms. Analysed n denotes the sample contributing to the review-selected outcome at the selected assessment, or the trial’s intention-to-treat sample where applicable. Analysed n may therefore equal randomised n despite loss to follow-up. NR = not reported. Abbreviations: BBMW, Breath–Body–Mind Workshop; BSRS-5, five-item Brief Symptom Rating Scale; DASS-21, Depression Anxiety Stress Scales-21; IBD, inflammatory bowel disease; ITT, intention-to-treat; MBSR, mindfulness-based stress reduction; NSCLC, non-small-cell lung cancer; PA, physical activity; PANAS, Positive and Negative Affect Schedule; PSS, Perceived Stress Scale; PSQ, Perceived Stress Questionnaire; RT, resistance training; STAI, State–Trait Anxiety Inventory; WEMWBS, Warwick–Edinburgh Mental Well-Being Scale.

**Table 2:** Subgroup analyses for the combined intervention versus passive or general control conditions.

Moderator	Category	*k*	Hedges’ *g* (95% CI)	*I*^2^ (%)	*p* for Subgroup Difference
Delivery format	Simultaneous	6	−0.46 (−0.77 to −0.16)	12.4	0.135
	Separate	6	−1.07 (−2.08 to −0.07)	86.9	
Study-level mean age	<40 years	6	−0.72 (−1.11 to −0.33)	24.4	0.905
	≥40 years	6	−0.77 (−1.87 to 0.33)	92.8	
Intervention duration	<8 weeks	7	−0.65 (−1.03 to −0.28)	31.6	0.641
	≥8 weeks	5	−0.89 (−2.26 to 0.48)	93.5	
Participant health status	Healthy/nonclinical	4	−0.71 (−0.94 to −0.47)	0.0	0.635
	Subclinical/high-stress	2	−0.96 (−3.88 to 1.97)	25.9	
	Clinical condition	6	−0.66 (−1.80 to 0.48)	92.1	
Physical-activity modality	Mind–body/yoga/tai chi	5	−0.63 (−1.17 to −0.08)	49.9	0.747
	Walking/general exercise	5	−0.85 (−2.25 to 0.55)	93.0	
	Stretching/resistance training	2	−0.85 (−2.77 to 1.07)	0.0	
Comparator type/intensity	Inactive/minimal controls	6	−0.65 (−1.04 to −0.26)	47.3	0.765
	Active/general controls	6	−0.79 (−1.89 to 0.32)	90.5	

Note. *p* values refer to formal between-subgroup tests. A statistically significant pooled effect within one category and a non-significant pooled effect within another category do not, by themselves, establish a statistically significant difference between subgroups.

## Data Availability

No new participant-level data were created in this systematic review. All data analysed were extracted from the published studies cited in this article. The study-level extraction dataset and analysis code are available from the corresponding author upon reasonable request.

## Supplementary Materials

The following supporting information can be downloaded at https://www.mdpi.com/article/10.3390/bs16091630/s1.

## References

[B1-behavsci-16-01630] Bohlmeijer E., Prenger R., Taal E., Cuijpers P. (2010). The effects of mindfulness-based stress reduction therapy on mental health of adults with a chronic medical disease: A meta-analysis. Journal of Psychosomatic Research.

[B2-behavsci-16-01630] Borenstein M., Hedges L. V., Higgins J. P., Rothstein H. R. (2009). Introduction to meta-analysis.

[B3-behavsci-16-01630] Cohen S., Janicki-Deverts D., Miller G. E. (2007). Psychological stress and disease. JAMA.

[B4-behavsci-16-01630] Creswell J. D., Lindsay E. K., Villalba D. K., Chin B. (2019). Mindfulness training and physical health: Mechanisms and outcomes. Psychosomatic Medicine.

[B5-behavsci-16-01630] DerSimonian R., Laird N. (1986). Meta-analysis in clinical trials. Controlled Clinical Trials.

[B6-behavsci-16-01630] Duval S., Tweedie R. (2000). Trim and fill: A simple funnel-plot-based method of testing and adjusting for publication bias in meta-analysis. Biometrics.

[B7-behavsci-16-01630] Egger M., Smith G. D., Schneider M., Minder C. (1997). Bias in meta-analysis detected by a simple, graphical test. BMJ.

[B8-behavsci-16-01630] Fischer J. M., Kandil F.-I., Kessler C. S., Nayeri L., Zager L. S., Rocabado Hennhöfer T., Steckhan N., Koppold-Liebscher D. A., Bringmann H. C., Schäfer T., Michalsen A., Jeitler M. (2022). Stress reduction by yoga versus mindfulness training in adults suffering from distress: A three-armed randomized controlled trial including qualitative interviews (RELAX study). Journal of Clinical Medicine.

[B9-behavsci-16-01630] Galbraith R. F. (1988). A note on graphical presentation of estimated odds ratios from several clinical trials. Statistics in Medicine.

[B10-behavsci-16-01630] Gerbarg P. L., Jacob V. E., Stevens L., Bosworth B. P., Chabouni F., DeFilippis E. M., Warren R., Trivellas M., Patel P. V., Webb C. D., Harbus M. D., Christos P. J., Brown R. P., Scherl E. J. (2015). The effect of breathing, movement, and meditation on psychological and physical symptoms and inflammatory biomarkers in inflammatory bowel disease: A randomized controlled trial. Inflammatory Bowel Diseases.

[B11-behavsci-16-01630] Goyal M., Singh S., Sibinga E. M. S., Gould N. F., Rowland-Seymour A., Sharma R., Berger Z., Sleicher D., Maron D. D., Shihab H. M., Ranasinghe P. D., Linn S., Saha S., Bass E. B., Haythornthwaite J. A. (2014). Meditation programs for psychological stress and well-being: A systematic review and meta-analysis. JAMA Internal Medicine.

[B12-behavsci-16-01630] Guyatt G., Oxman A. D., Akl E. A., Kunz R., Vist G., Brozek J., Norris S., Falck-Ytter Y., Glasziou P., DeBeer H., Jaeschke R., Rind D., Meerpohl J., Dahm P., Schünemann H. J. (2011). GRADE guidelines: 1. Introduction—GRADE evidence profiles and summary of findings tables. Journal of Clinical Epidemiology.

[B13-behavsci-16-01630] Hedges L. V., Olkin I. (1985). Statistical methods for meta-analysis.

[B14-behavsci-16-01630] Higgins J. P. T., Altman D. G., Gøtzsche P. C., Jüni P., Moher D., Oxman A. D., Savović J., Schulz K. F., Weeks L., Sterne J. A. C., Cochrane Bias Methods Group, Cochrane Statistical Methods Group (2011). The Cochrane Collaboration’s tool for assessing risk of bias in randomised trials. BMJ.

[B15-behavsci-16-01630] Higgins J. P. T., Thompson S. G., Deeks J. J., Altman D. G. (2003). Measuring inconsistency in meta-analyses. BMJ.

[B16-behavsci-16-01630] Hilcove K., Marceau C., Thekdi P., Larkey L., Brewer M. A., Jones K. (2021). Holistic nursing in practice: Mindfulness-based yoga as an intervention to manage stress and burnout. Journal of Holistic Nursing.

[B17-behavsci-16-01630] Huang J., Wei X., Yao L., Chi X., Xu W. (2024). Effects of combined mindfulness and physical activity intervention on depressive symptoms: A systematic review and meta-analysis. Mindfulness.

[B18-behavsci-16-01630] Hunt M., Al-Braiki F., Dailey S., Russell R., Simon K. (2018). Mindfulness training, yoga, or both? Dismantling the active components of a mindfulness-based stress reduction intervention. Mindfulness.

[B19-behavsci-16-01630] IntHout J., Ioannidis J. P. A., Borm G. F. (2014). The Hartung–Knapp–Sidik–Jonkman method for random effects meta-analysis is straightforward and considerably outperforms the standard DerSimonian–Laird method. BMC Medical Research Methodology.

[B20-behavsci-16-01630] IntHout J., Ioannidis J. P. A., Rovers M. M., Goeman J. J. (2016). Plea for routinely presenting prediction intervals in meta-analysis. BMJ Open.

[B21-behavsci-16-01630] Jain S., Shapiro S. L., Swanick S., Roesch S. C., Mills P. J., Bell I., Schwartz G. E. (2007). A randomized controlled trial of mindfulness meditation versus relaxation training: Effects on distress, positive states of mind, rumination, and distraction. Annals of Behavioral Medicine.

[B22-behavsci-16-01630] Kivimäki M., Steptoe A. (2018). Effects of stress on the development and progression of cardiovascular disease. Nature Reviews Cardiology.

[B23-behavsci-16-01630] Lin Latt C. M., Alldredge C. T., Williams S., Vinson M., Seiba Moris J., Elkins G. R. (2024). Mindful self-hypnosis combined with resistance training to reduce perceived stress and improve other psychological factors in female college students. International Journal of Clinical and Experimental Hypnosis.

[B24-behavsci-16-01630] Norouzi E., Naseri A., Rezaie L., Bender A. M., Salari N., Khazaie H. (2024). Combined mindfulness-based stress reduction and physical activity improved psychological factors and sleep quality in patients with MDD: A randomized controlled trial study. Archives of Psychiatric Nursing.

[B25-behavsci-16-01630] Page M. J., McKenzie J. E., Bossuyt P. M., Boutron I., Hoffmann T. C., Mulrow C. D., Shamseer L., Tetzlaff J. M., Akl E. A., Brennan S. E., Chou R., Glanville J., Grimshaw J. M., Hróbjartsson A., Lalu M. M., Li T., Loder E. W., Mayo-Wilson E., McDonald S., Moher D. (2021). The PRISMA 2020 statement: An updated guideline for reporting systematic reviews. BMJ.

[B26-behavsci-16-01630] Pedersen B. K., Saltin B. (2015). Exercise as medicine: Evidence for prescribing exercise as therapy in 26 different chronic diseases. Scandinavian Journal of Medicine & Science in Sports.

[B27-behavsci-16-01630] Polaski A. M., Phelps A. L., Smith T. J., Helm E. R., Morone N. E., Szucs K. A., Kostek M. C., Kolber B. J. (2021). Integrated meditation and exercise therapy: A randomized controlled pilot of a combined nonpharmacological intervention focused on reducing disability and pain in patients with chronic low back pain. Pain Medicine.

[B28-behavsci-16-01630] Reha N., Reha A. R. (2024). A brief yoga- and mindfulness-based psychoeducation program for women who have experienced domestic violence: An RCT study. Journal of Family Violence.

[B29-behavsci-16-01630] Remskar M., Western M. J., Osborne E. L., Maynard O. M., Ainsworth B. (2024). Effects of combining physical activity with mindfulness on mental health and wellbeing: Systematic review of complex interventions. Mental Health and Physical Activity.

[B30-behavsci-16-01630] Rotter G., Ortiz M., Binting S., Tomzik J., Reese F., Roll S., Brinkhaus B., Teut M. (2022). Mindful walking in patients with chronic low back pain: A randomized controlled trial. Journal of Integrative and Complementary Medicine.

[B31-behavsci-16-01630] Shi L., Welsh R. S., Lopes S., Rennert L., Chen L., Jones K., Zhang L., Crenshaw B., Wilson M., Zinzow H. (2019). A pilot study of mindful walking training on physical activity and health outcomes among adults with inadequate activity. Complementary Therapies in Medicine.

[B32-behavsci-16-01630] Singh B., Olds T., Curtis R., Dumuid D., Virgara R., Watson A., Szeto K., O’Connor E., Ferguson T., Eglitis E., Miatke A., Simpson C. E. M., Maher C. (2023). Effectiveness of physical activity interventions for improving depression, anxiety and distress: An overview of systematic reviews. British Journal of Sports Medicine.

[B33-behavsci-16-01630] Sterne J. A., Gavaghan D., Egger M. (2000). Publication and related bias in meta-analysis: Power of statistical tests and prevalence in the literature. Journal of Clinical Epidemiology.

[B34-behavsci-16-01630] Tang Y.-Y., Hölzel B. K., Posner M. I. (2015). The neuroscience of mindfulness meditation. Nature Reviews Neuroscience.

[B35-behavsci-16-01630] Terzioğlu Z. A., Çakır-Çelebi S. G. (2025). A brief online program integrating mindfulness and stretching exercises: Effects on well-being in health sciences students. Journal of Clinical Psychology.

[B36-behavsci-16-01630] Viechtbauer W. (2005). Bias and efficiency of meta-analytic variance estimators in the random-effects model. Journal of Educational and Behavioral Statistics.

[B37-behavsci-16-01630] Wang Y.-L., Zhang X.-F., Wang X.-P., Zhang Y.-J., Jin Y.-Y., Li W.-L. (2024). Combined mindfulness-based stress reduction and exercise intervention for improving psychological well-being in patients with non-small cell lung cancer. Clinical Psychology & Psychotherapy.

[B38-behavsci-16-01630] Xu X., Abdullah B. B., Samsudin S. B., Tan Y. (2025). Integrating mindfulness and physical activity: A meta-analysis of mindful movement interventions for symptoms of anxiety and depression among university students. PeerJ.

[B39-behavsci-16-01630] Zhang J., Qin S., Zhou Y., Meng L., Su H., Zhao S. (2018). A randomized controlled trial of mindfulness-based Tai Chi Chuan for subthreshold depression adolescents. Neuropsychiatric Disease and Treatment.

[B40-behavsci-16-01630] Zhang J.-Y., Li S.-S., Meng L.-N., Zhou Y.-Q. (2022). Effectiveness of a nurse-led mindfulness-based Tai Chi Chuan (MTCC) program on posttraumatic growth and perceived stress and anxiety of breast cancer survivors. European Journal of Psychotraumatology.

[B41-behavsci-16-01630] Zou L., Sasaki J. E., Wei G.-X., Huang T., Yeung A. S., Neto O. B., Chen K. W., Hui S. S.-c. (2018). Effects of mind–body exercises (Tai Chi/yoga) on heart rate variability parameters and perceived stress: A systematic review with meta-analysis of randomized controlled trials. Journal of Clinical Medicine.

