# Supplementary material for: The Combined Effect of Mindfulness and Physical Activity on Stress: A Systematic Review and Meta-Analysis of Randomized Controlled Trials
Source: Behav Sci (Basel). 2026 Sep 11;16(9):1630. doi: 10.3390/bs16091630 (PMC13603683; doi:10.3390/bs16091630)
Supplement: Supplementary file 1 [file behavsci-16-01630-s001.zip › behavsci-4398573-supplementary.pdf]

Supplementary Materials: Tables S1–S3 and Figures S1–S12

Supplementary Table S1. Database-specific search strategies

Search period: November 2025

Database / source	Fields / syntax	Search period	Complete search strategy	Records retrieved	Limits / notes
PubMed	Title/Abstract; MeSH; Publication Type	November 2025	(<br>(mindful*[Title/Abstract] OR "Mindfulness"[MeSH])<br>AND<br>(<br>yoga[Title/Abstract] OR "Yoga"[MeSH] OR<br>"tai chi"[Title/Abstract] OR "Tai Ji"[MeSH] OR<br>qigong[Title/Abstract] OR "Qi Gong"[MeSH] OR<br>"mindful walk*"[Title/Abstract] OR "walking meditation"[Title/Abstract] OR<br>"mindful movement"[Title/Abstract] OR<br>"physical activit*"[Title/Abstract] OR "Exercise"[MeSH] OR<br>"aerobic exercise"[Title/Abstract] OR<br>"mind-body therap*"[Title/Abstract] OR "mind-body intervention*"[Title/Abstract] OR<br>"Mind-Body Therapies"[MeSH]<br>)<br>)<br>AND<br>(<br>stress[Title/Abstract] OR "Stress, Psychological"[MeSH] OR<br>anxiety[Title/Abstract] OR "Anxiety"[MeSH] OR<br>depress*[Title/Abstract] OR "Depression"[MeSH] OR<br>"psychological distress"[Title/Abstract]<br>)<br>AND<br>(<br>intervention*[Title/Abstract] OR<br>"randomized controlled trial"[Publication Type] OR<br>"randomised controlled trial"[Title/Abstract] OR<br>RCT[Title/Abstract] OR<br>"Clinical Trial"[Publication Type]<br>)	2117	No language filter.

Web of Science Core Collection	Topic (TS)	November 2025	TS=(mindful* AND (yoga OR "tai chi" OR qigong OR "mindful walk*" OR "walking meditation" OR "mindful movement" OR "physical activit*" OR exercise OR "aerobic exercise" OR "mind-body therap*" OR "mind-body intervention*"))<br>AND TS=(stress OR anxiety OR depress* OR "psychological distress")<br>AND TS=("intervention*" OR "randomized controlled trial*" OR "randomised controlled trial*" OR RCT)	3088	No language filter.
Scopus	TITLE-ABS-KEY	November 2025	TITLE-ABS-KEY(("mindful yoga" OR "mindfulness-based yoga" OR "mindful walking" OR "mindful exercise" OR ("mindful*" AND (yoga OR "tai chi" OR qigong OR "walking meditation"))) AND (stress OR "perceived stress" OR "Perceived Stress Scale" OR PSS) AND ("randomized controlled trial" OR "randomised controlled trial" OR RCT)) AND PUBYEAR > 1999	512	Publication year > 1999; no language filter.
EBSCOhost	TI, AB, SU	November 2025	(TI AB SU: (mindfulness OR mindful-based OR "mindfulness-based intervention" OR MBI OR MBSR OR "mindfulness meditation" OR "mindful training")) AND (TI AB SU: ("physical activity" OR exercise OR "physical exercise" OR "aerobic exercise" OR "resistance training" OR sport OR "movement intervention" OR "physical training")) AND (TI AB SU: (stress OR "psychological stress" OR "perceived stress" OR "stress level" OR "stress symptom" OR distress)) AND (TI AB SU: ("combined intervention" OR "combined program" OR "dual intervention" OR "integrated intervention" OR co-intervention)) AND (TI AB SU: ("randomized controlled trial" OR RCT OR intervention OR pretest posttest OR clinical trial)) NOT (TI AB SU: (animal OR rat OR mouse OR monkey OR rodent))	1334	Search string reproduced as run; no language filter.
ProQuest Dissertations & Theses Global	Keyword/proximity search (NEAR/3)	November 2025	(("mindfulness-based" OR mindful*) NEAR/3 (yoga OR "tai chi" OR qigong OR "mindful walk*" OR "walking meditation" OR "mindful movement" OR "physical activit*" OR exercise OR "aerobic exercise")) AND (stress OR "perceived stress" OR "stress reactivity" OR "psychological stress") AND ("randomized controlled trial" OR "randomised controlled trial" OR RCT OR "clinical trial" OR "experimental study") AND PY >= 2000	238	Publication year >= 2000. Unpublished dissertations/theses were ineligible under the peer-reviewed publication criterion.
ScienceDirect	TITLE-ABS-KEY (as executed)	November 2025	TITLE-ABS-KEY((mindfulness OR MBSR) AND (exercise OR yoga) AND stress AND RCT)	151	Exact syntax confirmed by the authors; no language filter.
JSTOR	General keyword search	November 2025	"mindful yoga" OR "mindfulness-based yoga" OR "mindful walking" OR "mindful exercise"	84	No language filter.
Springer Nature Link	General keyword search	November 2025	(mindfulness OR MBSR) AND ("physical activity" OR exercise) AND (stress OR "perceived stress" OR psychological distress) AND ("combined intervention" OR integrated program) AND ("randomized controlled trial" OR RCT) AND human NOT animal	430	No language filter.

**Total database records identified**				**7,954**	Four additional unique records were identified through reference-list screening and citation searching.

Language: No language filter was applied during database searching; reports published in English or Chinese were eligible for inclusion.

Note: Search strategies were adapted to the indexing systems, searchable fields, interface functions, and Boolean-search capabilities of individual databases. The breadth and combination of search terms therefore varied across databases; where more complex combinations resulted in overly restrictive retrieval, simpler combinations were used to avoid excessive loss of potentially relevant records. The strategies shown above represent the actual searches conducted in each database.

Supplementary Table S2. Measurement instruments, selected outcomes, assessment time points, and effect-size data sources for included trials

Fourteen trials involving 872 randomised participants were included in the systematic review; 13 trials contributed to at least one comparator-specific quantitative synthesis.

Study	Randomised n	Analysed n	Selected construct	Instrument / version	Scoring direction	Selected assessment
Gerbarg et al. (2015)	29	25 (13/12) for PSQ at week 6	Perceived stress	Perceived Stress Questionnaire (PSQ)	Higher scores indicate greater perceived stress	Week 6
Polaski et al. (2021)	52	38 (18/20)	State anxiety	State-Trait Anxiety Inventory, Form Y, state subscale (STAI-Y)	Higher scores indicate greater anxiety	Exit assessment, 48 h after the 4-week intervention
Wang et al. (2024)	60	60 (30/30)	General psychological distress	Five-item Brief Symptom Rating Scale (BSRS-5)	Higher scores indicate greater distress	Week 8
Terzioğlu and Çakır-Çelebi (2025)	44	38 (19/19)	Mental well-being	Warwick-Edinburgh Mental Well-Being Scale (WEMWBS)	Higher scores indicate better well-being	Immediate post-test after session 6
Reha and Reha (2024)	59	51 (27/24)	Stress symptoms	Depression Anxiety Stress Scales-21, stress subscale (DASS-21 Stress)	Higher scores indicate greater stress	Post-test after 5 weeks
Shi et al. (2019)	38	37 (16/21)	Perceived stress	Perceived Stress Scale (PSS; version not reported)	Higher scores indicate greater perceived stress	Immediate post-intervention assessment (4 weeks)
Zhang et al. (2018)	64	62 (32/30)	Perceived stress	Chinese Perceived Stress Scale (CPSS)	Higher scores indicate greater perceived stress	Post-intervention after 8 weeks
Zhang et al. (2022)	59	58 (29/29) at immediate post-test	Perceived stress	Perceived Stress Scale (PSS)	Higher scores indicate greater perceived stress	Immediate post-intervention assessment after 8 weeks
Hilcove et al. (2021)	80	78 (41/37)	Perceived stress	Perceived Stress Scale (PSS-10)	Higher scores indicate greater perceived stress	Post-intervention after 6 weeks
Fischer et al. (2022)	102	102 in ITT (35/33/34)	Perceived stress	German Perceived Stress Scale (PSS-10)	Higher scores indicate greater perceived stress	V1, 12 weeks/post-intervention
Norouzi et al. (2024)	67	67 (16/17/17/17)	Perceived stress	Farsi Perceived Stress Scale (PSS-14)	Higher scores indicate greater perceived stress	8-week post-test
Lin Latt et al. (2024)	44	30 (11/8/11)	Perceived stress	Perceived Stress Scale (PSS-10)	Higher scores indicate greater perceived stress	Post-test after 5 weeks

Study	Randomised n	Analysed n	Selected construct	Instrument / version	Scoring direction	Selected assessment
Hunt et al. (2018)	119	110 completed the post-treatment stress challenge	State anxiety, affect, and stress reactivity	STAI state subscale; PANAS; HRV/SDNN	Direction varies by measure	Weekly/session assessments and post-treatment stress challenge
Rotter et al. (2022)	55	48 available-case PSS analyses at week 8	Perceived stress	Cohen's Perceived Stress Scale (PSS-14)	Higher scores indicate greater perceived stress	Week 8

Supplementary Table S2 (continued). Comparator and effect-size data source

Study	Comparator(s)	Effect-size data source / reconstruction	Direction handling	Quantitative-synthesis status
Gerbarg et al. (2015)	Time-matched IBD educational seminar	Unadjusted post-intervention group means, SDs, and analysed n	No sign reversal	Included: passive/general control
Polaski et al. (2021)	Audiobook active/general control	Unadjusted post-intervention group means, SDs, and analysed n	No sign reversal	Included: passive/general control
Wang et al. (2024)	Routine health education and psychological nursing	Unadjusted post-intervention group means, SDs, and analysed n	No sign reversal	Included: passive/general control
Terzioğlu and Çakır-Çelebi (2025)	No-treatment control	Unadjusted post-intervention group means, SDs, and analysed n	Sign reversed so negative Hedges' g favours the combined intervention	Included: passive/general control
Reha and Reha (2024)	Wait-list control	Unadjusted post-intervention group means, SDs, and analysed n	No sign reversal	Included: passive/general control
Shi et al. (2019)	Physical-activity advice plus Fitbit	Adjusted change contrast; standardised using pooled baseline SD; SE approximated from reported two-sided p = 0.02; generic inverse-variance entry	Direction aligned so negative values favour the combined intervention	Included: passive/general control; reconstructed estimate
Zhang et al. (2018)	Usual physical-education curriculum	Unadjusted post-intervention group means, SDs, and analysed n	No sign reversal	Included: passive/general control
Zhang et al. (2022)	Wait-list control	Unadjusted post-intervention group means, SDs, and analysed n	No sign reversal	Included: passive/general control
Hilcove et al. (2021)	No-intervention control	Unadjusted post-intervention group means, SDs, and analysed n	No sign reversal	Included: passive/general control
Fischer et al. (2022)	Iyengar yoga; mindfulness training alone	Post-intervention data from the intention-to-treat analysis	No sign reversal	Included: physical-activity-alone and mindfulness-alone comparisons
Norouzi et al. (2024)	Physical activity alone; MBSR alone; sertraline control	Unadjusted post-intervention group means, SDs, and analysed n	No sign reversal	Included in all three comparator-specific analyses
Lin Latt et al. (2024)	Resistance training alone; wait-list control	Unadjusted post-intervention group means, SDs, and analysed n	No sign reversal	Included: passive/general control and physical-activity-alone comparisons
Hunt et al. (2018)	Mindfulness alone; yoga alone; active placebo; no treatment	Exact group-level variance information was insufficient for a reproducible effect-size estimate; no unreported values were imputed	Not applicable	Systematic review only; excluded from quantitative synthesis

Study	Comparator(s)	Effect-size data source / reconstruction	Direction handling	Quantitative-synthesis status
Rotter et al. (2022)	No-intervention control	Baseline-adjusted endpoint mean difference (-1.6; 95% CI -4.8 to 1.6) converted to SE, standardised using pooled baseline SD, and entered by generic inverse variance	Direction aligned so negative values favour the combined intervention	Included: passive/general control; reconstructed estimate

Note. Randomised n denotes the number allocated to study arms. Analysed n denotes the sample contributing to the review-selected outcome at the selected assessment, or the trial's intention-to-treat sample where applicable. Negative Hedges' g values were defined as favouring the combined intervention. Shi et al. (2019) and Rotter et al. (2022) used reconstructed estimates entered by generic inverse variance. Hunt et al. (2018) was retained in the systematic review but excluded from quantitative synthesis because a reproducible effect-size variance could not be obtained.

Supplementary Table S3. GRADE assessment of the certainty of evidence for the three comparator-specific primary analyses

Comparison	Studies	Participants, n	Risk of bias	Inconsistency	Indirectness	Imprecision	Publication bias	Hedges' g (95% CI)	Certainty
Combined intervention vs passive or general control conditions	12 RCTs	550	Serious[1]	Serious[2]	Not serious	Not serious	Not serious[3]	−0.74 (−1.21 to −0.27)	LOW ⨁⨁◯◯
Combined intervention vs mindfulness alone	2 RCTs	102	Serious[1]	Not serious[4]	Not serious	Very serious[5]	Not serious[3]	0.49 (−1.71 to 2.69)	VERY LOW ⨁◯◯◯
Combined intervention vs physical activity alone	3 RCTs	120	Serious[1]	Not serious[4]	Not serious	Serious[6]	Not serious[3]	0.01 (−0.50 to 0.52)	LOW ⨁⨁◯◯

[1] Risk of bias: The evidence was downgraded because contributing trials included unclear or high-risk judgements in domains relevant to subjective outcomes, particularly allocation concealment and blinding of participants/personnel or outcome assessment.

[2] Inconsistency: The passive/general-control comparison showed substantial heterogeneity ($I^2 = 83.1\%$), and the 95% prediction interval crossed the null.

[3] Publication bias: No downgrade was applied. In the passive/general-control comparison, Egger's regression did not indicate statistically significant small-study effects and trim-and-fill imputed no missing studies, although power was limited. The mindfulness-alone and physical-activity-alone comparisons contained too few studies for informative formal assessment.

[4] Inconsistency: No downgrade was applied, but between-study inconsistency could not be meaningfully evaluated because only two and three studies contributed to the mindfulness-alone and physical-activity-alone comparisons, respectively.

[5] Imprecision: Downgraded two levels because only two trials were available and the confidence interval was very wide, including appreciable effects in either direction as well as the null.

[6] Imprecision: Downgraded one level because only three trials were available and the confidence interval crossed the null and included potentially important effects in either direction.

Note. GRADE = Grading of Recommendations Assessment, Development and Evaluation; RCT = randomised controlled trial; CI = confidence interval. The outcome was the stress or stress-related psychological outcome selected for the review at the earliest eligible post-intervention assessment. Negative Hedges' g values favour the combined intervention. Randomised trials start at high certainty and may be downgraded for risk of bias, inconsistency, indirectness, imprecision, and publication bias. The GRADE assessment was conducted during manuscript revision in response to reviewer comments and was not part of the retrospectively registered review plan. Participants, n, refers to participants contributing to the review-selected outcome at the assessment time point used in each comparator-specific meta-analysis.

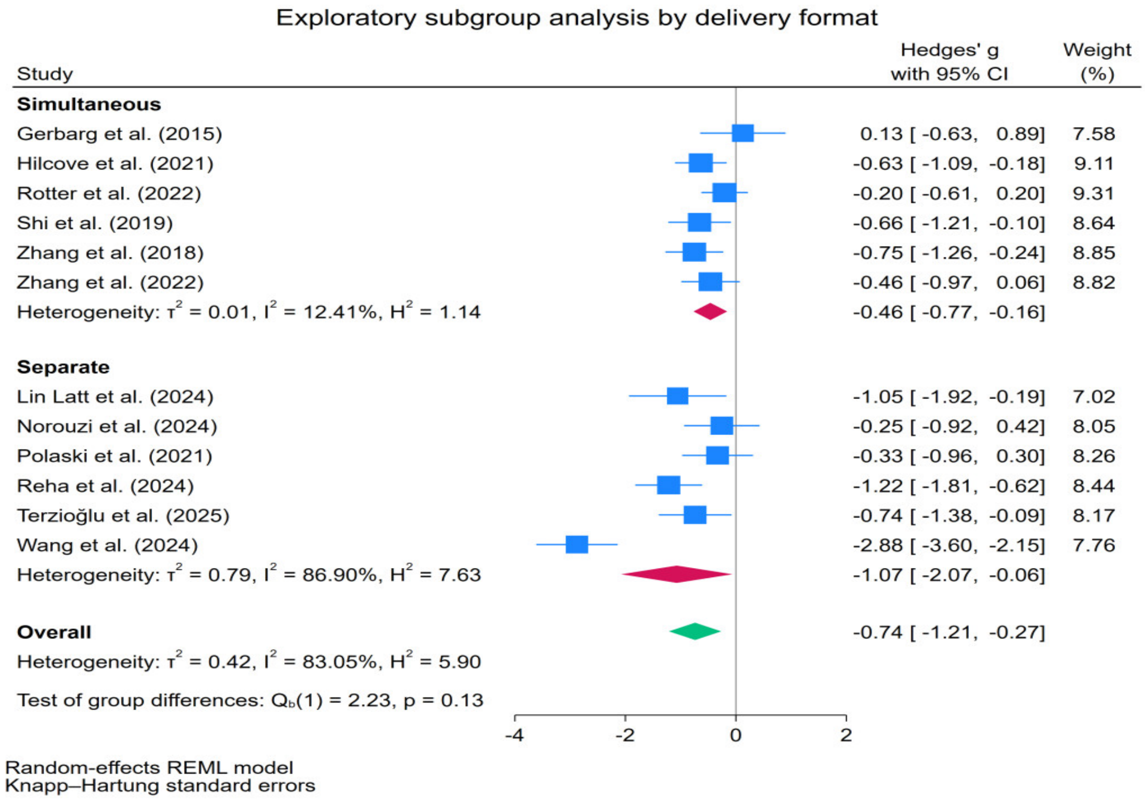

Supplementary Figure S1 delivery subgroup

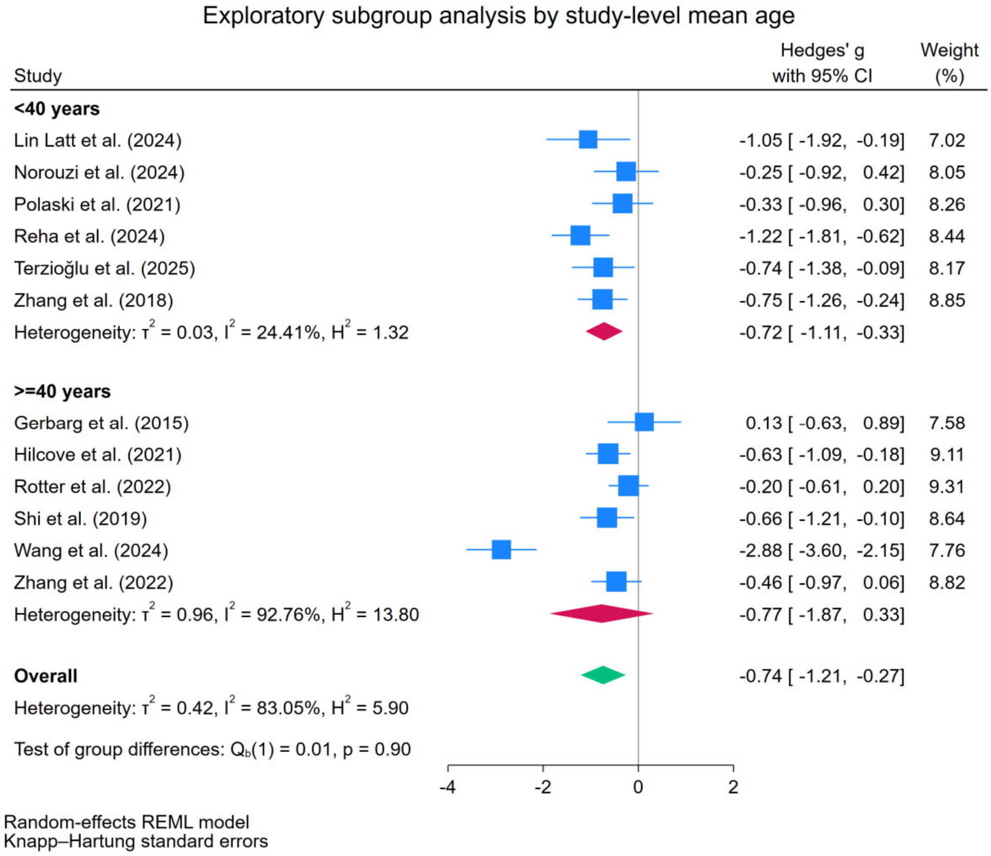

Supplementary Figure S2 age subgroup

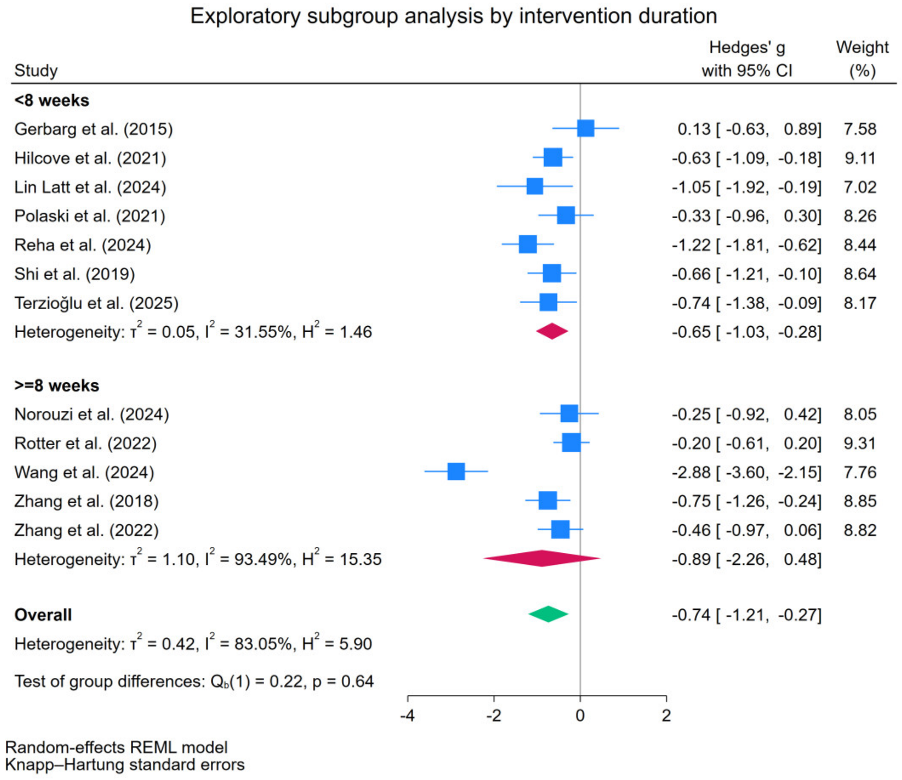

Supplementary Figure S3 duration subgroup

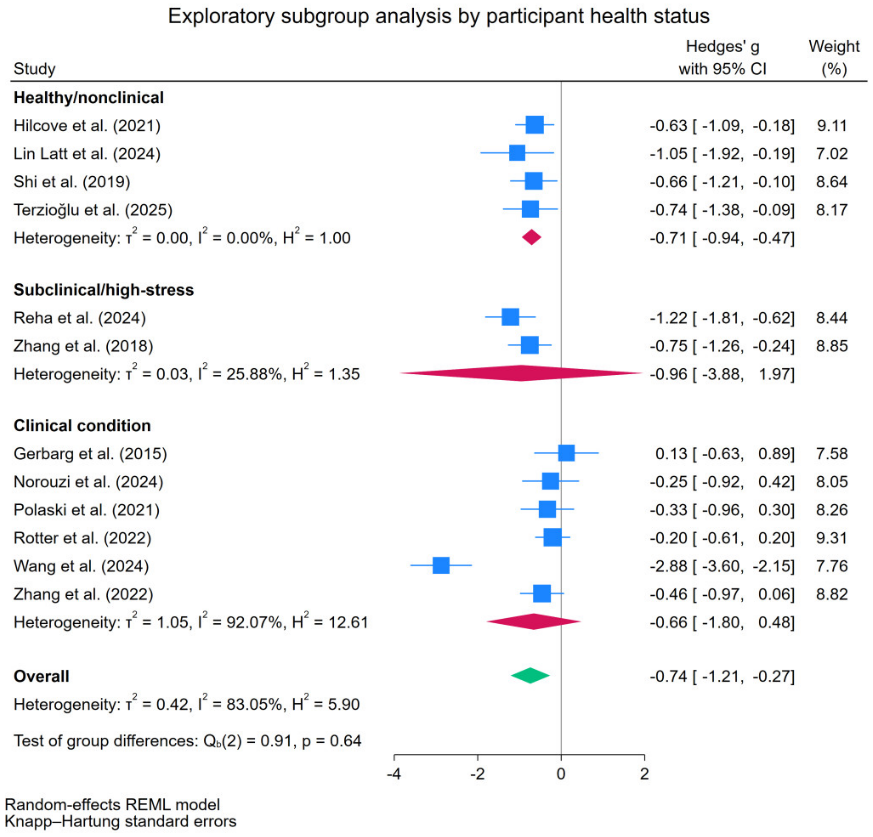

Supplementary Figure S4 health status subgroup

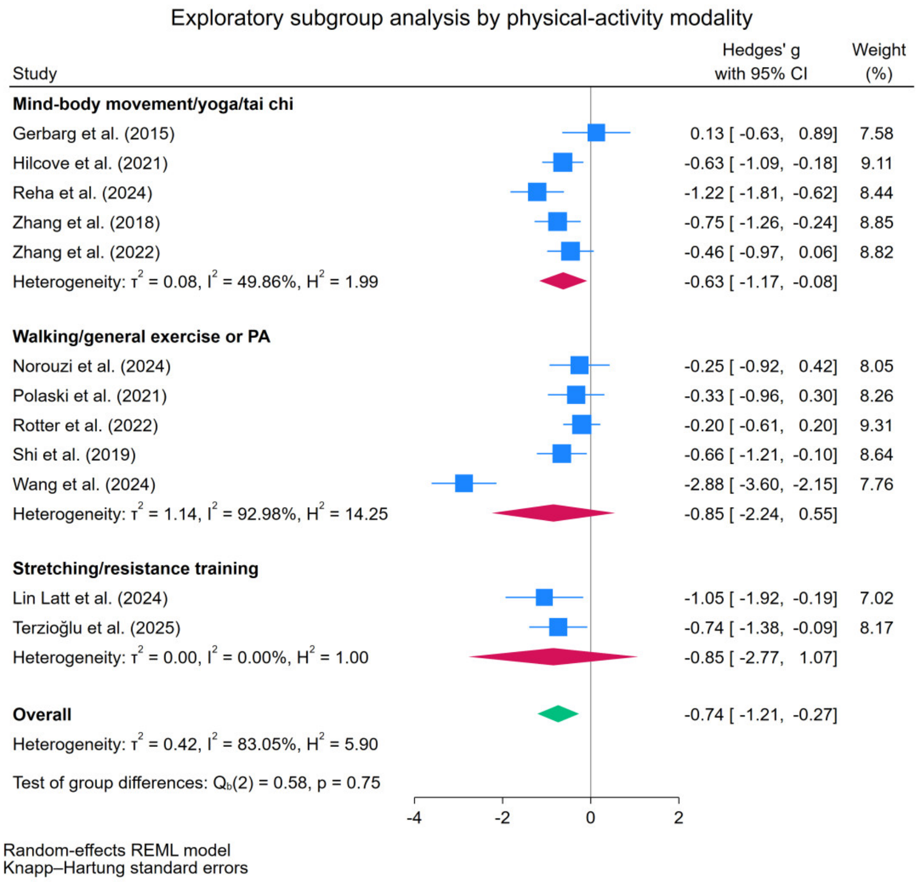

Supplementary Figure S5 PA modality subgroup

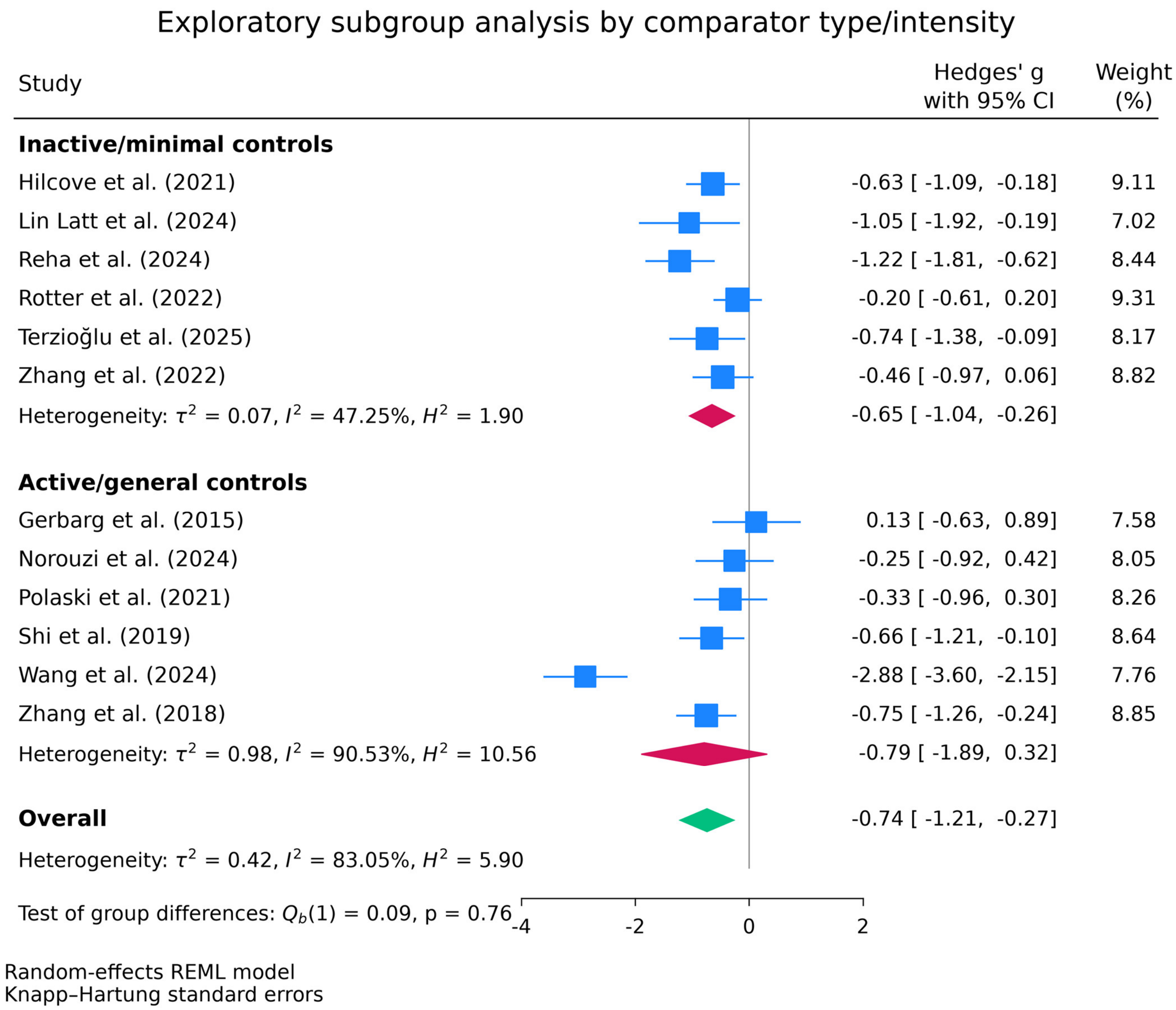

Supplementary Figure S6 comparator type/intensity subgroup

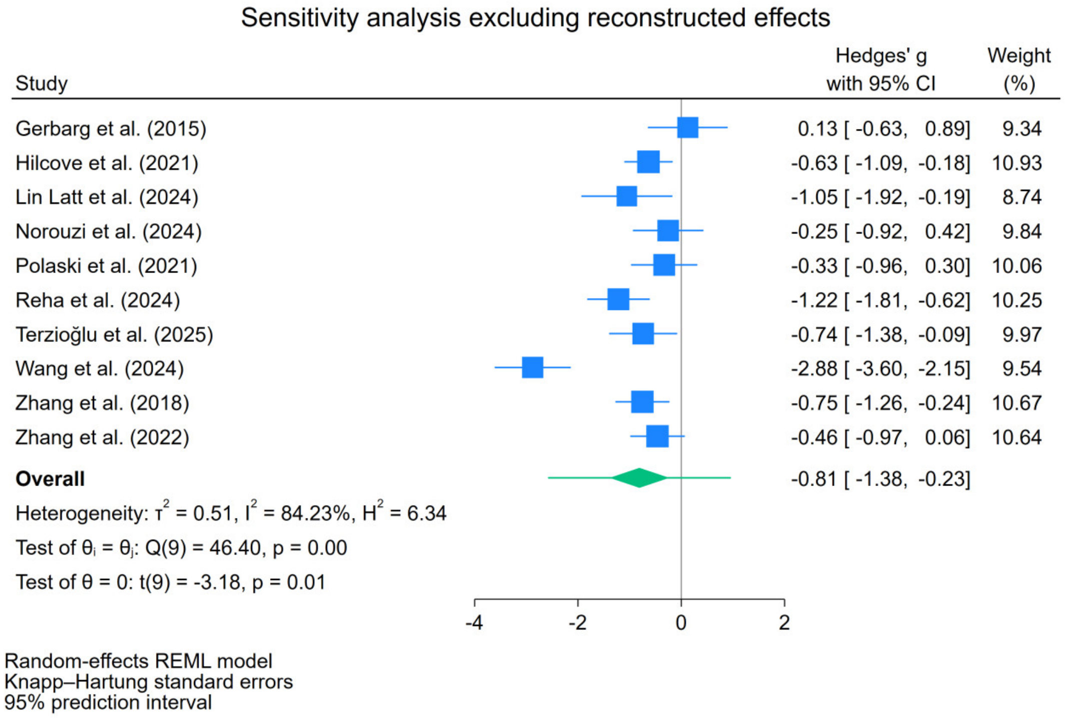

Supplementary Figure S7 excluding reconstructed effects

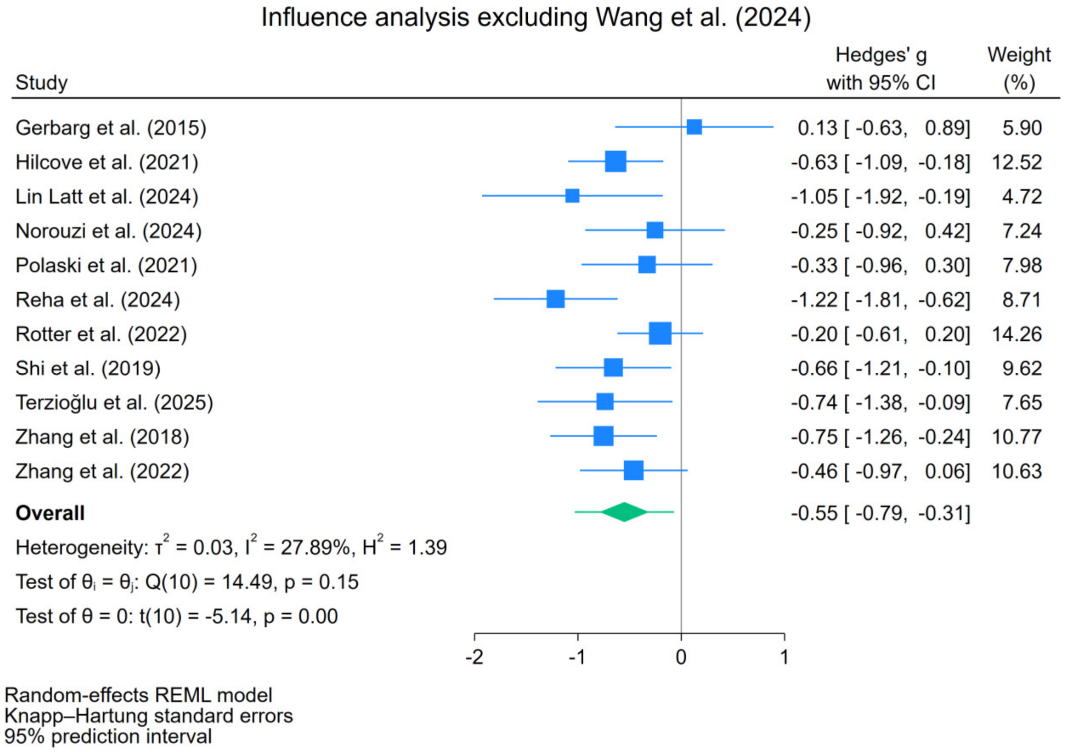

Supplementary Figure S8 excluding Wang

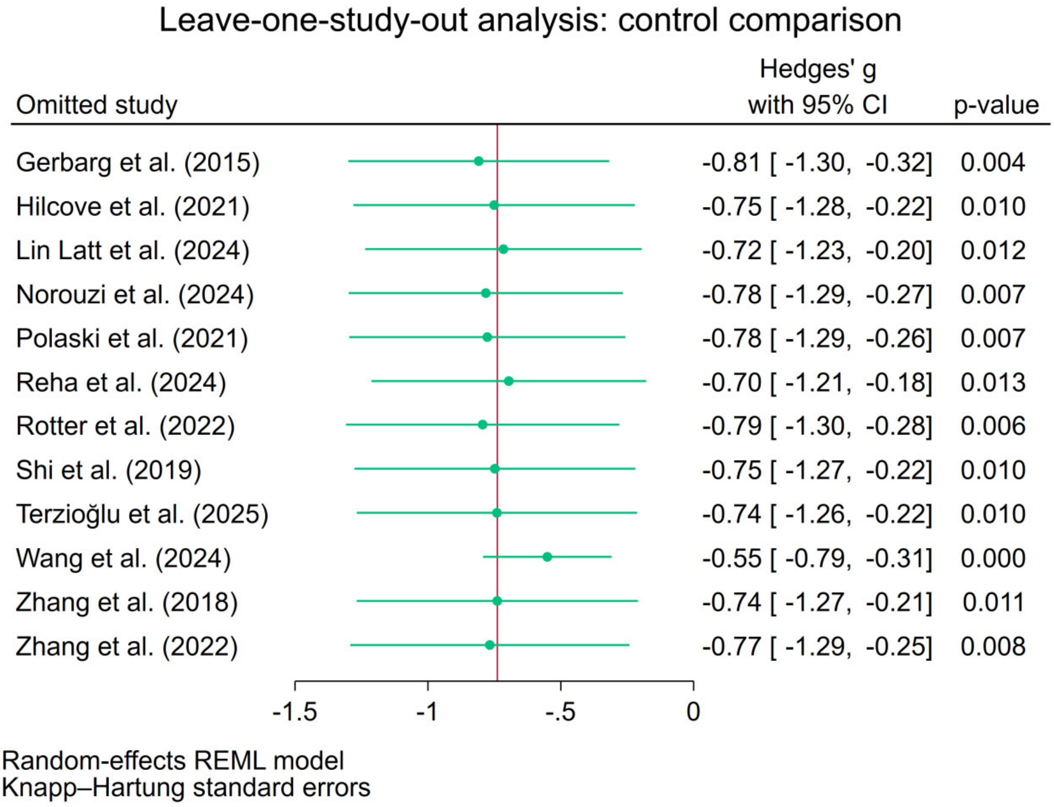

Supplementary Figure S9 leave one study out

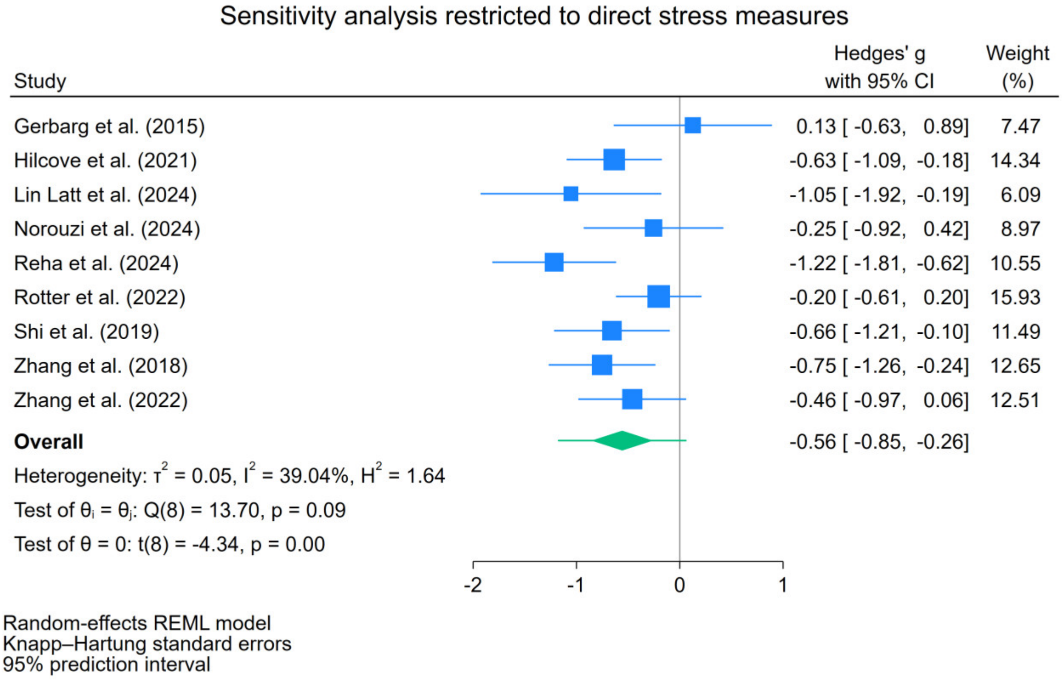

Supplementary Figure S10 direct stress measures

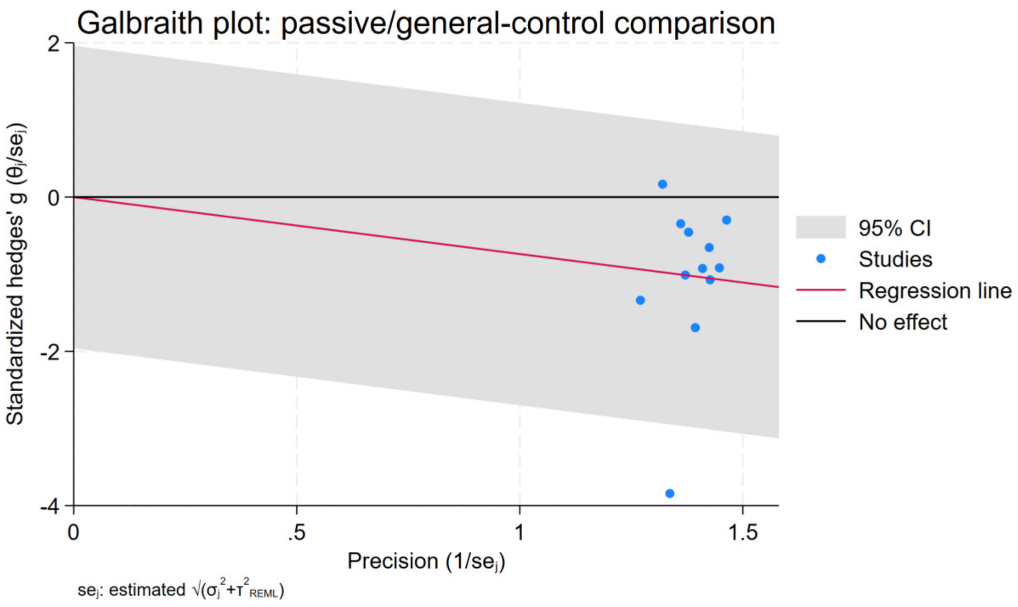

Supplementary Figure S11 Galbraith plot

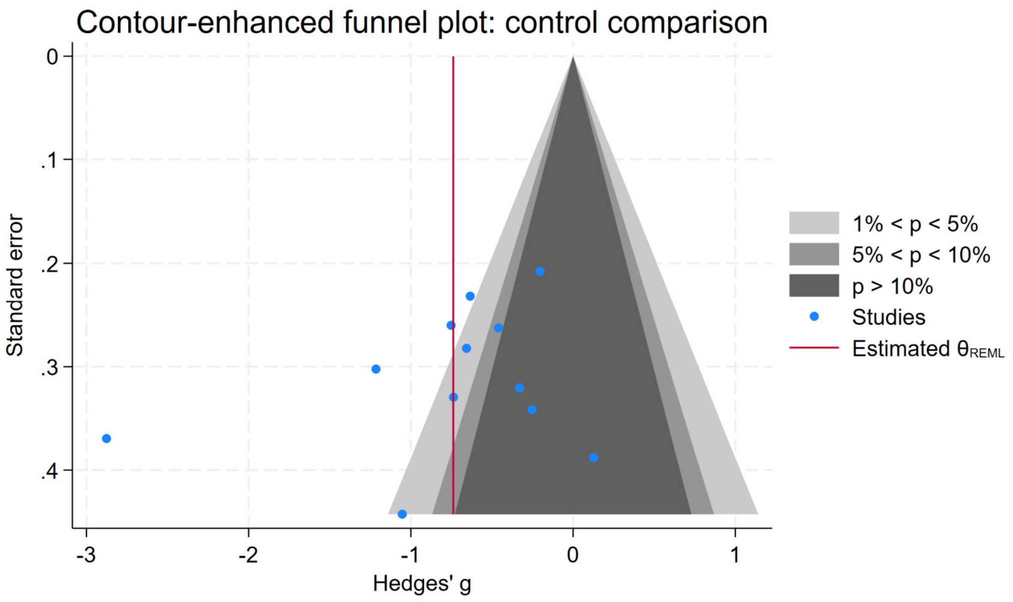

Supplementary Figure S12 contour enhanced funnel
